# Adipose‐Derived Mesenchymal Stem Cells: Isolation, Origin, Phenotype Applications, and Challenges

**DOI:** 10.1002/mco2.71103

**Published:** 2026-11-03

**Authors:** Ziyang Liu, Guangming Tu, Chao Wang, Hongyan Zhao, Ziyi Wu, Wei Li, Li Chen, Bailin Niu

**Affiliations:** ^1^ Department of Plastic Surgery, Chongqing Emergency Medical Center Chongqing University Central Hospital, School of Medicine, Chongqing University Chongqing China; ^2^ Department of Intensive Care Medicine, Chongqing Emergency Medical Center Chongqing University Central Hospital, School of Medicine, Chongqing University Chongqing China; ^3^ Department of Cardiothoracic Surgery, Chongqing Emergency Medical Center Chongqing University Central Hospital, School of Medicine, Chongqing University Chongqing China; ^4^ Department of Intensive Care Medicine Chongqing Hospital, Union Hospital, Tongji Medical College, Huazhong University of Science and Technology Chongqing China

**Keywords:** adipose‐derived mesenchymal stem cells (AD‐MSCs), cellular heterogeneity, extracellular vesicles (EVs), functional subpopulations, targeted preconditioning

## Abstract

Traditional adipose‐derived mesenchymal stem cells (AD‐MSCs) research has relied on unfractionated bulk populations, whose therapeutic efficacy is constrained by cellular heterogeneity, culture‐induced phenotypic drift, and rapid postengraftment functional exhaustion, resulting in inconsistent outcomes and impeding clinical translation. This review systematically examines AD‐MSC developmental origins, tissue niches, isolation and purification strategies, functional phenotypes, and biomanufacturing bottlenecks. It integrates preclinical and clinical evidence for AD‐MSCs and their extracellular vesicles (EVs) in bone and cartilage regeneration, chronic cutaneous diseases, neurological disorders, cardiovascular diseases, and other conditions. These therapies remodel injured microenvironments through immunometabolic reprogramming, antifibrotic signaling, angiogenesis, mitochondrial transfer, and paracrine regulation rather than direct tissue replacement. Major translational barriers include cellular heterogeneity, safety concerns, the transdifferentiation controversy, nonstandardized preconditioning, culture‐induced phenotypic drift, and low cell retention after transplantation. We propose that clinical translation should be based on prospective isolation of functional subpopulations, coupled with tailored preconditioning, precision gene editing, three‐dimensional biomaterials, EV engineering, and automated closed‐system biomanufacturing. This framework aims to preserve potency, phenotypic stability, and paracrine function, transforming AD‐MSCs and their bioactive derivatives from variable, unfractionated preparations into well‐characterized, standardized, highly potent precision biologics. More broadly, it offers a mechanism‐driven, function‐defined, engineering‐enabled, and clinically accessible roadmap for next‐generation regenerative medicine.

## Graphical abstract

Bridging basic biology to clinical translation, this review systematically explores the in vivo origins and evolving isolation techniques of AD‐MSCs. We dissect their diverse phenotype applications orchestrated by immunometabolic reprogramming, mitochondrial transfer, and extracellular vesicles. Integrating precise preconditioning, EV bioengineering, and 3D biomaterials can maximize core regenerative mechanisms, ultimately transforming AD‐MSCs into standardized clinical therapies. This figure was created at BioRender.com.

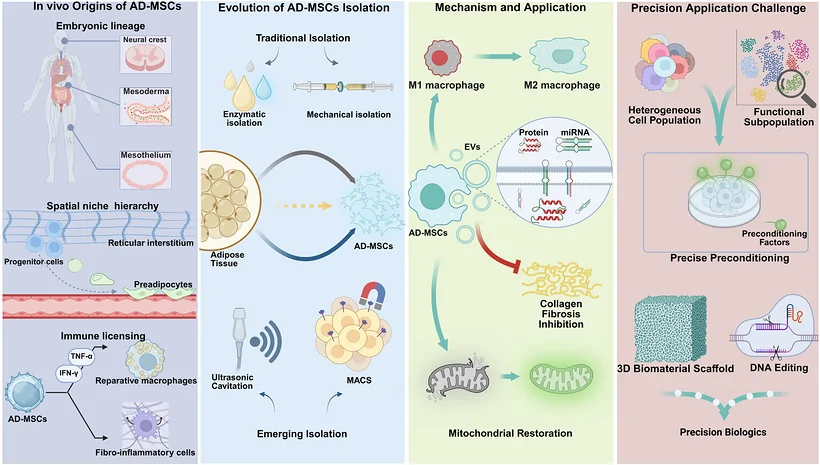

## 1 Introduction

Adipose tissue is currently recognized as an energy reservoir, a dynamic and valuable endocrine organ, and a rich source of stem cells for regenerative medicine. Compared with traditional bone marrow‐derived mesenchymal stem cells (BM‐MSCs), adipose‐derived mesenchymal stem cells (AD‐MSCs) are easier to collect, are more abundant, and exhibit multilineage plasticity alongside immunomodulatory properties [1, 2]. When strategically combined with biomaterials, they orchestrate microenvironmental allostasis to effectively repair damaged tissues and organs in clinical settings [3].

With respect to the history of AD‐MSCs (Figure 1), Owen and Friedenstein conceptualized the use of the marrow stromal stem cell system in 1988 [4]. This study laid the groundwork for Arnold Caplan's formal introduction of the concept of mesenchymal stem cells (MSCs) in 1991 [5]. In 2001, Zuk et al. [6] pioneered the isolation of multipotent adherent cells from human liposuction waste. These cells were initially termed processed lipoaspirate cells (PLAs). As they share a phenotype with BM‐MSCs but are easily harvested in large quantities under local anesthesia, these cells redefine adipose tissue from a metabolic burden into an accessible regenerative core. The International Federation for Adipose Therapeutics and Science (IFATS) and the International Society for Cellular Therapy (ISCT) issued a joint statement in 2013 standardizing the phenotypic and functional criteria for the uncultured stromal vascular fraction (SVF) and in vitro‐expanded AD‐MSCs to address the conflicting nomenclature (e.g., PLA and ADAS) [7]. Crucially, early translational frameworks erroneously assumed that MSCs exert therapeutic effects via direct structural transdifferentiation in vivo. However, due to the pervasive absence of definitive *Cre‐loxP* lineage‐tracing evidence, subsequent analyses revealed that transplanted MSCs undergo rapid apoptotic clearance within harsh, ischemic recipient microenvironments, and exhibit negligible long‐term somatic persistence [8, 9]. From a clinical perspective, the 2016 approval of Alofisel for complex perianal fistulas was a major milestone. It officially ushered allogeneic AD‐MSCs into the realm of standardized biologics [10]. Over time, however, the in vivo pharmacokinetic data revealed a discrepancy. Despite the initial hopes, these transplanted cells showed almost no long‐term structural engraftment. Given the insurmountable allostatic barriers preventing true transdifferentiation, in 2017, Caplan fundamentally redefined MSCs as medicinal signaling cells [11]. This name change marked a major conceptual shift in the field. AD‐MSCs do not heal damaged tissue through a physical replacement. Instead, their therapeutic efficacy relies almost entirely on a transient paracrine licensing mechanism. Because transplanted living cells are rapidly eliminated by blood‐mediated inflammatory reactions and recipient cytotoxic networks [12], this research organically focused on the isolation of their functional derivatives. Consequently, extracellular vesicles (EVs) have emerged as a major research hotspot. As nanosized intercellular signaling mediators, EVs encapsulate the regenerative molecular payload of AD‐MSCs while effectively circumventing physical pulmonary microvascular entrapment, thermodynamic instability, and the risk of allorejection associated with intact living cells [13]. Between 2018 and 2019, *Nature* and *Science* published single‐cell transcriptomic atlases of adipose tissue, identifying highly specialized mesenchymal subpopulations, such as highly proliferative, multipotent DPP4^+^ interstitial progenitors [14, 15], and redirecting regenerative medicine toward the precise targeting of functional cell subpopulations.

**FIGURE 1:**
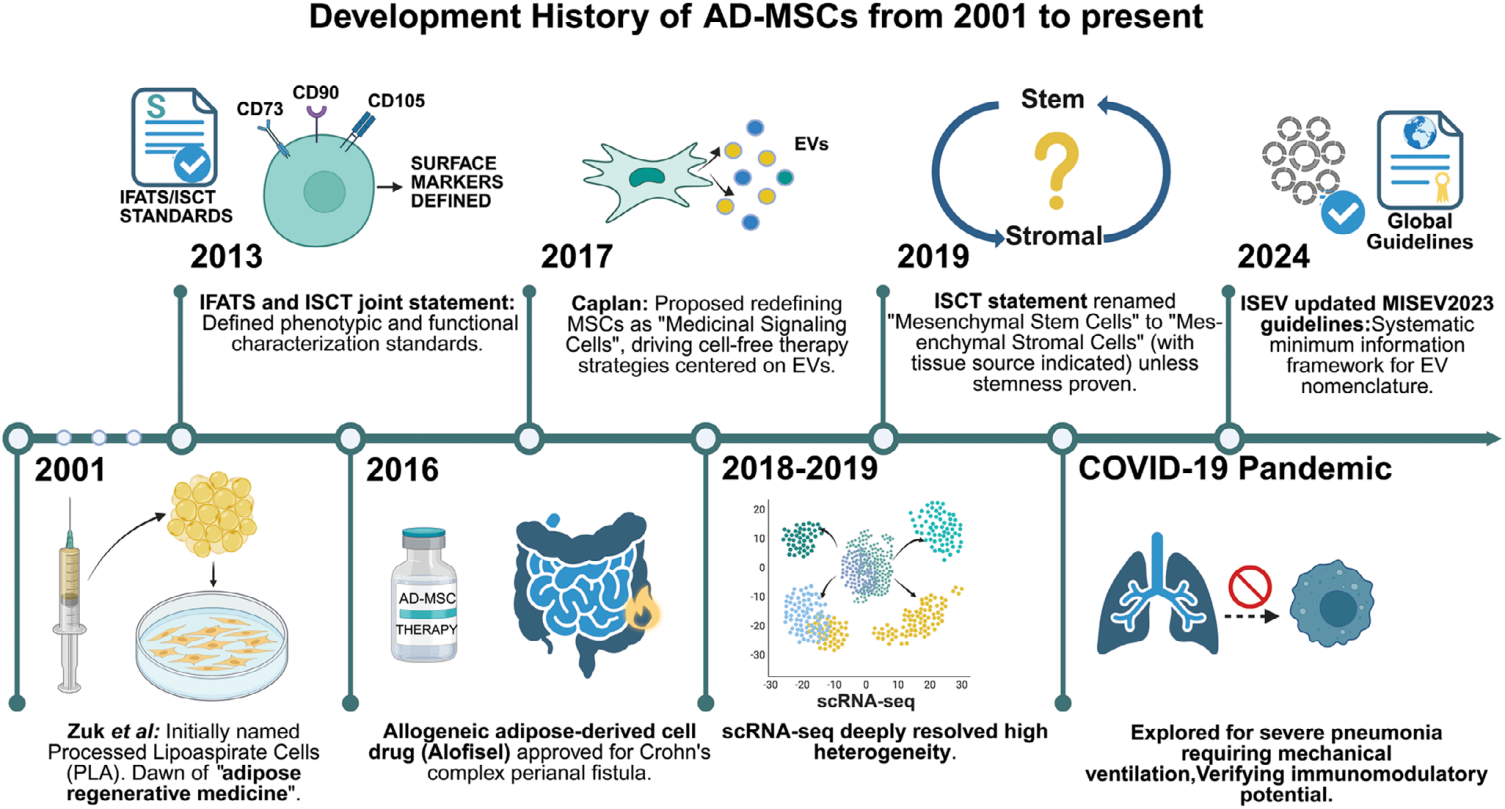
Chronological milestones in the research and clinical translation of AD‐MSCs. The timeline depicts the developmental trajectory of AD‐MSC therapies over the past two decades. This figure was created at BioRender.com. IFATS, International Federation for Adipose Therapeutics and Science; ISCT, International Society for Cellular Therapy; ISEV, International Society for Extracellular Vesicles.

The 2019 ISCT statement recommended renaming “mesenchymal stem cells” to “mesenchymal stromal cells” to further standardize the terminology unless rigorous in vitro and in vivo experiments proved stemness, and the statement advocated for tissue‐specific prefixes (e.g., AD‐MSCs) [16]. The immunomodulatory potential of AD‐MSCs in severe inflammatory microenvironments was explored during the COVID‐19 pandemic. Early clinical trials provided an exploratory proof‐of‐concept, indicating that intravenous infusions of AD‐MSCs in critically ill patients with pneumonia could safely attenuate clinical symptoms and downregulate inflammatory markers [17]. In parallel to cellular therapies, the rapid expansion of cell‐free strategies prompted the International Society for Extracellular Vesicles (ISEV) to release the MISEV2023 guidelines in 2024, establishing rigorous criteria for EV isolation and characterization to ensure the rigor, reproducibility, and transparency of EV‐based therapeutics [18].

Currently, AD‐MSCs and their derived EVs are widely applied in multiple fields because of their pleiotropic plasticity and immunomodulatory effects [19, 20, 21, 22, 23, 24, 25, 26, 27, 28]. Numerous related clinical treatments have rapidly emerged in recent years. Their therapeutic efficacy can be further augmented through targeted preconditioning regimens, such as hypoxia [29, 30]. Even with these scientific milestones, a significant translational gap remains. Previously, many studies simply reported positive observational outcomes. Unfortunately, these studies rarely examined the underlying biological flaws that drive inconsistent clinical results. Today, several severe bottlenecks continue to obstruct the widespread clinical adoption of AD‐MSCs and mainly include unstandardized biomanufacturing protocols, culture‐induced phenotypic drift, and the rapid allostatic exhaustion of unstratified cell populations immediately after engraftment [31, 32, 33, 34, 35, 36]. Therefore, the central thesis of this review is that overcoming the current stagnation of the translation of AD‐MSCs necessitates the abandonment of monolithic bulk‐cell paradigms. Precision regenerative medicine mandates the structural identification, isolation, and targeted preconditioning of distinct functional subpopulations.

Our review presents a practical roadmap to address this conceptual gap. Ultimately, we aim to advance both the foundational biology and the clinical translation of AD‐MSCs. We begin with a critical appraisal of the evolutionary trajectory of methods for isolating AD‐MSCs, alongside optimized strategies for in vitro culture that safeguard cellular identity, potency and batch‐to‐batch consistency. We then examine the EV‐mediated cell‐free therapeutic paradigm, an emerging framework that is reshaping the mechanistic and translational landscape of AD‐MSC‐based regenerative medicine. Moving from therapeutic modalities to the underlying biology, we next evaluate organ‐specific regenerative mechanisms driven by AD‐MSCs and their bioactive derivatives across a diverse array of disease models. We conclude by confronting the three core translational bottlenecks limiting AD‐MSC deployment: cellular heterogeneity, safety liabilities, and suboptimal homing efficiency. We propose targeted preconditioning of functionally defined cell subpopulations as a pathway toward standardized, clinical‐grade regenerative biologics.

## 2 Developmental Origins and Bioprocessing of AD‐MSCs

This section outlines biological and technical considerations for AD‐MSCs, tracing their path from the origins in the native tissue toward industrial biomanufacturing. We first review the in vivo ontogeny, spatial hierarchy, and immune‐licensing mechanisms that intrinsically govern the therapeutic boundaries of these progenitors. We next shift to ex vivo processing and critically evaluate established and novel isolation approaches by comparing enzymatic, mechanical, magnetic, and ultrasonic techniques for the isolation of pure subpopulations that are compliant with advanced therapeutic medicinal product (ATMP) regulations. We further examine how the biochemical composition of the media, 3D culture configuration, and hypoxia gradient shape transcriptomic stability and senescence during cell expansion. The section closes by identifying key biological effectors that are released following cell isolation. Collectively, this sequential analysis, spanning developmental biology to controlled bioprocessing, defines the technical prerequisites required to convert heterogeneous adipose tissue fractions into standardized biological products.

### 2.1 In Vivo Origins of AD‐MSCs

Defining the therapeutic utility of AD‐MSCs requires that researchers first decipher their developmental ontogeny and tissue niches. Clinical translation has historically encountered bottlenecks when the adipose SVF is treated as a monolithic progenitor reservoir. Recent single‐cell and spatial transcriptomic atlases have demonstrated that multilineage plasticity and therapeutic efficacy are largely determined by embryonic lineage specification, spatial localization, and reciprocal immune licensing [37, 38].

#### 2.1.1 Embryonic Lineage and Epigenetic Memory

Embryonic ontogeny involves persistent epigenetic imprinting that determines the functional trajectory of AD‐MSCs. Lineage‐tracing models confirm that craniofacial adipose depots originate from the neural crest, which confers a greater neurogenic transdifferentiation capacity on these stem cells. In contrast, trunk depots originate from distinct mesodermal lineages. Subcutaneous progenitors derived from paraxial and lateral plate mesoderm carry epigenetic marks that support hyperplastic expansion and systemic immunomodulation. Visceral progenitors, in contrast, emerge from mesothelium‐associated tissues. These cells are predisposed to elevated basal lipolytic activity and easily undergo proinflammatory polarization under metabolic stress [38]. Accordingly, researchers should carefully match the embryonic source of MSCs to the specific disease environment. This principle provides the theoretical basis for harnessing depot‐specific stem cell phenotypes.

#### 2.1.2 Tissue Niche and Mesenchymal Hierarchy

Precise spatial coordinates within adipose tissue tightly shape regenerative cellular behavior. Advanced intersectional lineage tracing overturns the traditional view that equates these stem cells exclusively with pericytes and establishes adventitial fibroblasts as the major regenerative progenitor population [38].

Spatial transcriptomics is used to map a continuous developmental hierarchy within the perivascular niche. Highly proliferative CD55‐positive and PI16‐positive interstitial progenitors are at the top of this hierarchy. These uncommitted cells are present in the reticular interstitium, actively maintain microenvironmental homeostasis, and exert strong immunomodulatory effects. As they commit to becoming adipocytes, they migrate toward the vasculature. During this differentiation process, their transcriptomic profiles gradually converge, eventually generating APOD‐positive preadipocytes [37]. Clinical therapies should prioritize the use of progenitors at the top of the hierarchy to maintain stable cellular phenotypes, given that downstream committed cells are more susceptible to stress‐induced senescence.

#### 2.1.3 Immune Licensing of AD‐MSCs

Autologous stem cell therapies often yield unpredictable results, especially when the cells are harvested from donors with metabolic comorbidities. Explaining this variability remains a critical translational challenge. The inflammatory licensing concept posits that these stem cells require transient stimulation by proinflammatory cues, including interferon‐gamma and tumor necrosis factor‐alpha, to trigger metabolic reprogramming. Once activated, stem cells secrete immunosuppressive effectors that drive local macrophages toward a reparative polarization state [39]. Recipient microenvironments characterized by chronic inflammation or age‐associated changes impose substantial homeostatic stress that disrupts this reciprocal regulation. Under this pathological stress, certain groups of progenitor cells make the wrong developmental choices. Rather than following regenerative trajectories, they deviate into profibrotic fibroinflammatory progenitors or age‐associated regulatory cells. This pathological conversion drives excessive extracellular matrix (ECM) deposition and initiates paracrine inhibitory effects, abolishing tissue repair. Researchers must prospectively isolate specific progenitor subpopulations at the top of the hierarchy that are resistant to profibrotic phenotypes to ensure consistent therapeutic outcomes in patients with severe clinical conditions.

### 2.2 Techniques for Isolating AD‐MSCs

The clinical translation of AD‐MSCs is fundamentally limited by the use of extraction and ex vivo propagation methods. Beyond their preparative utility, these bioprocessing procedures impose microenvironmental stress that dictates the transcriptomic stability and regulatory classification of the cellular product. Standardizing these procedures, primarily enzymatic digestion, mechanical dissociation, MACS, and ultrasound‐mediated cavitation, is essential for bridging the translational gap. Each strategy yields distinct outcomes, which are defined by its degree of structural disruption and compliance with ATMP regulatory frameworks (Table 1).

**TABLE 1:** Comparison of AD‐MSCs isolation methods.

Method		Technical strategy	Yield	Isolation time	Product purity	Surface marker expression	Cell characteristics	Differentiation time	Advantages	Disadvantages
	Coleman fat (control) [40]	Centrifuge fat aspirates at 1200 × *g* for 3 min. Remove the upper oil layer and lower water layer without applying shear force.	/	3 min	/	/	Contains intact, mature adipocytes. Significantly more total living cells than that in the experimental group.	/	Simple preparation process. Maintains a relatively high number of total living cells without mechanical destruction.	A lower long‐term graft retention rate in vivo. Presence of residual oil drops in grafts.
	SVF/ECM gel [40]	Centrifuge at 1200 × *g* for 3 min. Add 0.5 mL of oil, and mechanically shift the sample between syringes via a 2.4 mm connector for 1 min at 10 mL/s. Centrifuge at 2000 × *g* for 3 min.	The final volume is 25% that of the control. Increased cell density. No significant difference in the total SVF levels.	7 min	/	Greater than threefold increase in CD34 expression. Significant increases in CD13, CD73, CD146, CD45, and CD31 expression	Higher SVF cell density. Increased cell pluripotency. Formed a mature lobular fat structure with new adipocytes.	Same as the control	Pure physical action without enzymes. Removes oil drops. Enriches regeneration‐related cells. Improves fat retention in vivo.	Mechanical shear force destroys mature adipocytes, leading to a significantly decreased number of total living cells.
	FAT‐ED (control) [41]	Digest with 1 mg/mL Type I collagenase at 37°C for 45 min. Centrifuge at 400 × *g* for 10 min and treat with lysis buffer (10 min).	5 × 10^5^ ± 2 × 10^5^ nucleated cells/mL. CFU‐F yield: 4877 ± 2477/mL.	65 min	/	CD34^+^ (3.7%), CD105^+^ (10.0%), CD73^+^ (10.1%).	High proliferative activity and a short doubling time (45.3 ± 4.0 h). High CFU‐F frequency (1.7%).	Confluence is reached in 5 ± 2 days. (The doubling time is approximately 45 h).	Highly efficient cell release. Produces the greatest absolute number of free nucleated cells and CFU‐Fs.	Time consuming. Considered a substantially manipulated product using proteases (ATMP) and faces strict regulatory restrictions.
	Hy‐Tissue SVF [41]	Mechanical disaggregation. Massage fat for 5 min. Force through a 120 µm filter bag and centrifuge at 400 × *g* for 10 min.	4.1 × 10^4^ ± 2 × 10^4^ nucleated cells/mL. CFU‐F yield: 178 ± 49/mL.	15–20 min	/	CD34^+^ (9.9%, significantly higher than the control). After expansion (P1 generation), high expression of CD105/90/73/29/44 is detected.	Microfragments may provide more sustained paracrine effects. Enrichment of CD34^+^ cells.	Confluence is reached in 8 ± 2 days. (The doubling time is 59.4 ± 2.8 h). Tripartite differentiation: same as the control.	Simple, fast, and nonenzymatic method. Minimal manipulation avoids ATMP restrictions. Preserves natural tissue architecture.	Significantly lower nucleated free cell yield and CFU‐F yield compared with those of enzymatic digestion.
MACS	Direct adherence (AD‐MSC‐A) (control) [42]	Perform enzymatic digestion of the SVF, followed by direct adherence, culture, and passaging to passage 3 or 4.	High (highest overall cell yield among these three methods).	1 h	Sca‐1 rate: 60.47%	CD29: 81.40%. CD44, CD90, CD31, and CD45 show little difference in expression compared with the other groups.	Short, fusiform shape. Lowest proliferation rate, lowest CFU‐F capacity, and early signs of cellular aging.	CCK‐8 assay: 6 days. CFU‐F: 14 days. Adipogenic: 10.5 days (median). Osteogenic/chondrogenic differentiation: 21 days.	Produces the highest total cell yield. Simple, traditional, standard protocol.	Lowest cell purity. Highly susceptible to early culture contamination by cell debris and lipid droplets.
	MACS then adherence (AD‐MSC‐M) [42]	Perform enzymatic digestion of the SVF, immediate MACS of Sca‐1‐positive cells, then adherence culture.	Low (decreased by > 50% immediately after MACS; lowest overall yield).	1.5 h	Sca‐1 rate: 78.37%	CD29: 83.41%.	Short, fusiform shape. More uniform morphology and increased proliferation compared with AD‐MSC‐A.	Same as the control.	Eliminates Sca‐1‐negative cells early to improve purity compared to direct adherence.	Premature sorting causes substantial cell loss.
	Adherence then MACS (AD‐MSC‐AM) [42]	Perform enzymatic digestion of the SVF, culture to passage 3 to gain a growth advantage, followed by MACS of Sca‐1‐positive cells.	Moderate (initially similar to AD‐MSC‐A, decreased by ∼30% after sorting).	Several weeks	Highest Sca‐1 rate of 98.37%	CD29: 98.43%.	Homogeneous, 3D elongated spindle shape. Highest proliferative capacity and CFU‐F ability. Enrichment of angiogenesis and immune regulation functions.	Same as the control.	Best balance of yield and purity. Greatest adipogenic differentiation capacity. High proliferation capacity.	Susceptible to early culture contamination before sorting. The overall cell yield is lower than that of direct adherence.
	Ultrasound cavitation [43]	Chop lipoaspirates and expose to ultrasound (tested power from 0–6, time from 0 to 10 min). Irradiate at power 6 for 10 min, filter, and centrifuge.	No significant difference in yield compared to the control group. The output of thoroughly hand‐shredded material is approximately twice that obtained with a mechanical homogenizer.	13 min	/	CD90/29/105/73/13 expression levels were slightly higher than those in the control group, but the differences were not statistically significant.	Functional capabilities were perfectly preserved. Shows a nonsignificant trend of an increased CFU‐F count compared with the control group.	Same as the control group.	Chemical‐ and enzyme‐free. Significantly decreased isolation time. Avoids cGMP concerns related to enzyme usage. Maintains identical cell yields.	The yield mainly depends on manual preprocessing (tissue chopping yields ∼2 × more cells than a dissociator) and optimization of the ultrasound machine settings.

Abbreviation: MACS, magnetic‐activated cell sorting.

#### 2.2.1 Enzymatic Digestion

Enzymatic digestion involves the use of exogenous collagenases to proteolytically degrade the ECM, releasing SVF, which is a heterogeneous mixture of AD‐MSCs, endothelial progenitor cells, pericytes, fibroblasts, and immune cells [44]. This method yields high cell counts with approximately 10% of cells expressing CD34 (indicating endothelial cells and AD‐MSCs) and maintains multilineage plasticity, making it a routine method for basic research [41, 45].

However, global regulatory authorities classify exogenous enzymatic digestion coupled with ex vivo cell expansion as substantial manipulation, subjecting these populations to stringent ATMP classifications. This regulatory requirement mandates adherence to current good manufacturing practice (cGMP) protocols to systematically mitigate culture‐induced cellular stress, transcriptomic drift, and epigenetic alterations during scale‐up. Even within this demanding regulatory environment, adipose‐derived progenitors offer key practical advantages over bone marrow‐derived stem cells. Primary lipoaspirates naturally contain a high initial yield of MSCs. The inherently high baseline percentage of MSCs in primary lipoaspirates minimizes the number of population doublings necessary to achieve therapeutic doses. This growth advantage is crucial. It delays replicative senescence, protects genomic fidelity, and reduces the risk of clonal exhaustion, which often affects other stem cell sources during large‐scale industrial manufacturing [46, 47].

#### 2.2.2 Mechanical Dissociation

Mechanical dissociation has emerged as a rapid, cost‐effective, and simple‐to‐implement alternative that is well suited for rapid clinical applications. Although the initial cell viability decreases after shear stress, the recovered mesenchymal stromal cells maintain metabolic activity [40, 48, 49]. Crucially, mechanically derived fractions frequently satisfy regulatory thresholds for minimal manipulation. This advantage reduces the heavy costs of cGMP manufacturing constraints. It also prevents the epigenetic drift that typically occurs during prolonged laboratory culture. Nevertheless, this regulatory exemption comes with biological trade‐offs. Mechanical fragmentation inherently precludes the prospective isolation and targeted preconditioning of functional subsets. Deploying uncultured stromal fractions imposes intractable compositional heterogeneity [50].

#### 2.2.3 MACS

Immunomagnetic bead sorting technology enables efficient cell separation and purification by targeting markers expressed on the surface of AD‐MSCs [51]. In conjunction with this approach, sorting strategies that target specific stemness markers have been shown to further optimize cell quality. For instance, recent studies have indicated that adherent culture followed by magnetic bead sorting (AD‐MSC‐AM) targeting the murine‐specific marker stem cell antigen‐1 (Sca‐1) increases the purity of AD‐MSCs to more than 95%, specifically enriching a subpopulation characterized by superior proliferative kinetics, increased adipogenic differentiation potential, and augmented proangiogenic and immunomodulatory transcriptomic profiles [42]. The lack of a functional Sca‐1 homolog in humans limits the direct clinical translation of this sorting strategy. Clinical biomanufacturing should sort human cells effectively by enriching functional progenitor subsets based on the expression of defined marker panels. CD55 or PI16 identifies uncommitted progenitor cells located in the reticular interstitium. Isolating this population prevents premature lineage commitment during ex vivo expansion [37]. DPP4‐based selection results in the enrichment of a highly proliferative and multipotent interstitial subpopulation. The CD146 antigen is expressed by a specialized perivascular subset that exhibits specific immunomodulatory and proangiogenic functions [52]. Negative selection for CD142 depletes the highly procoagulant cellular fraction. Integrating this negative gating into the upstream isolation protocol establishes a baseline safety barrier for the cellular product. Implementing these human markers in large‐scale MACS faces notable physical constraints. Native human mesenchymal surface receptors are sensitive to fluid shear stress during continuous‐flow sorting and industrial fluidic processing. These mechanical forces induce the proteolytic cleavage and shedding of target surface receptors. This progressive antigen loss decreases antibody binding efficiency, limiting the yield and purity of the isolated human subsets. Future cell processing strategies could utilize genetic engineering to express stable animal orthologs on human AD‐MSCs and subsequently attenuate this receptor lability. Operating as shear‐stable surrogate sorting markers, these animal‐derived markers are less susceptible to mechanical shedding, standardizing the physical separation of human subpopulations during industrial biomanufacturing.

#### 2.2.4 Ultrasound‐Assisted Techniques

Ultrasound approaches offer versatile, nonenzymatic processing via two distinct physical mechanisms: high‐intensity cavitation for isolation and low‐intensity stimulation for preconditioning. Ultrasonic cavitation uses microbubble implosion to rapidly disrupt adipose tissue and separate cells without exogenous enzymes, reducing the risk of immune rejection and the processing time and abrogating ATMP‐associated enzymatic compliance issues [43]. AD‐MSCs isolated via cavitation show no significant differences in terms of cell yield or the expression of phenotypic markers compared with cells obtained using enzymatic digestion.

### 2.3 Ex Vivo Culture Environments of AD‐MSCs

The movement of AD‐MSCs from their natural perivascular niche into an artificial manufacturing culture system places them under substantial biological stress. Research in contemporary regenerative medicine aims to establish a unified mechanistic framework where biochemical formulations, spatial dimensionality, and oxygen gradients function synergistically as microenvironmental cues. These factors collectively shape the transcriptomic heterogeneity of AD‐MSCs, fundamentally reprogram their therapeutic secretome, and govern their senescence progression and multilineage differentiation potential prior to engraftment.

#### 2.3.1 The Influence of Biochemical Culture Conditions on AD‐MSCs

Culture media fundamentally shape the transcriptomic state of AD‐MSCs (Table 2). Relying on standard xenogeneic supplements, such as fetal bovine serum, may shift cells toward replicative exhaustion. This decline is evidenced by an increase in population doubling time from 23.53 h at early stages to 105.83 h at advanced passages, along with the upregulation of the senescence checkpoints CDKN1A and TP53 [53]. Researchers have adopted human platelet lysate to mitigate this limitation, which transiently shifts the transcriptomic profile toward an anti‐inflammatory state and prolongs the survival of septic murine models to 70.1% compared with 50.0% for mice treated with serum‐expanded cell populations [54]. However, pooled human blood products represent a notable barrier to clinical translation. Strict manufacturing specifications require pathogen reduction steps, which may result in the degradation of key lipid‐bound growth factors within the lysate. Combined with the intrinsic batch‐to‐batch variability of human blood materials, a consistent final product quality is difficult to maintain [55, 56].

**TABLE 2:** Assessment of AD‐MSC culture formulations.

Methods	Advantages	Disadvantages	Refs.
FBS media	Supports biological growth by providing a macromolecular crowding effect. Shows partial efficacy in downregulating specific systemic proinflammatory cytokines in acute sepsis models.	Induces significantly lower collagen synthesis and extracellular matrix (ECM) deposition than xeno‐free media due to the presence of matrix metalloproteinases. Prolonged exposure imposes an allostatic burden, driving cells toward replicative exhaustion.	[53, 54, 55, 56, 57, 61]
hPL	Abundantly rich in platelet‐derived growth factors, drastically enhancing MSC proliferation without compromising chromosomal stability or relative telomere length (RTL). Quantitatively rescues survival rates in acute inflammatory models to a greater extent than serum‐expanded populations.	Retains inherent risks of human blood‐borne viral transmission. Mandatory pathogen reduction (PR) technologies may negatively affect platelet metabolism and function. The absence of a universal global consensus on standardized production workflows and quantitative release criteria limits broad comparability. Inherently contains plasma‐borne isoagglutinins which may cause incompatibilities depending on the cell types.	[54, 55, 56]
DM/SFM	Their pure, rule‐compliant nature resolves allogeneic inconsistencies and standardizes manufacturing. Animal component‐free (ACF) and xeno‐free (XF) media enhance endogenous ECM deposition and multilineage differentiation capabilities compared to FBS. Specific optimized formulations mitigate oxidative and replicative senescence while restoring tissue homeostasis.	Artificial 2D liquid media inherently lack the native 3D macromolecular crowding (MMC) effect; external crowders must be supplemented to maximize tissue‐specific ECM deposition. Commercial CDM formulations are often proprietary and exceptionally cost‐prohibitive, restricting massive clinical expansion.	[56, 57, 58, 59, 60, 61]

Abbreviations: CDM, chemically defined media; ECM, extracellular matrix; FBS, fetal bovine serum; hPL, human platelet lysate; SFM, serum‐free media.

Transitioning to a chemically defined medium (CDM) or serum‐free medium (SFM) can mitigate these batch‐to‐batch variations. The assertion that serum‐free formulations accelerate proliferation and improve differentiation requires broader rigorous supporting evidence to validate the current limited findings. A properly tailored CDM [57] can result in an up to 24‐fold increase in the cell yield across a single passage while maintaining vital immune‐regulating functions, including indoleamine 2,3‐dioxygenase activity, intact [58]. Furthermore, serum‐free formulations actively attenuate senescence by suppressing IL‐6/STAT3 signaling, whereas calibrated basal compositions optimize bioenergetic homeostasis to compress the population doubling time to 29.21 h compared with 44.06 h for cells grown in conventional matrices, governing terminal osteogenic or chondrogenic differentiation without the confounding factors present in serum [59, 60].

Nevertheless, universal CDM implementation faces formidable mechanobiological limitations. In addition to requiring precise stoichiometric calibration to rescue blunted immunoregulatory functions [58], synthetic formulations lack the native macromolecular crowding effect observed in premium serum supplements. The attenuation of this excluded volume effect inherently impairs rheological mechanotransduction and restricts ECM deposition [61]. Current manufacturing workflows present a core trade‐off: CDM offers high purity and full regulatory compliance, while serum supplementation provides superior biological supportive capacity, and this challenge persists until synthetic crowding agents are fully optimized.

#### 2.3.2 The Effect of 3D Biophysical Confinement on AD‐MSCs

The physical properties of the culture environment profoundly modulate AD‐MSC transcriptomic heterogeneity and secretory profiles. Forced monolayer expansion on rigid planar substrates disrupts physiological mechanical homeostasis, increasing senescence‐associated β‐galactosidase activity. In contrast, culturing cells within soft 3D hydrogels that recapitulate the native tissue mechanics alleviates this senescent phenotype. Culturing cells in tissue‐mimetic hydrogels featuring a precisely calibrated stiffness of 3.7 kPa mitigates mechanical stress. This dimensional transition quantitatively decreases the senescent cell fraction from 11.5% to 3.6%, downregulates the mRNA expression of the senescence marker p16 by nearly threefold, and increases the secretion of regenerative EVs [62].

Despite maintaining bioenergetic homeostasis, spatial confinement alters the biological functions of AD‐MSCs. Physical crowding within hydrogels or hyperdense spatial seeding suppresses canonical adipogenic and mesenchymal programs. Transcriptomic profiling revealed that this physical crowding promotes the mesenchymal–epithelial transition (MET). This mechanotransduction cascade markedly downregulates the expression of classical mesenchymal markers, notably CD44 and CD105, while upregulating the expression of epithelial junctional proteins, including E‐cadherin, EpCAM, KRT6A, and MUC5B [63, 64].

#### 2.3.3 Hypoxic Modulation of AD‐MSC Metabolism and Differentiation

Finally, oxygen tension is a key factor regulating AD‐MSC potency. Ambient normoxia induces continuous oxidative stress, accelerating metabolic exhaustion, and precipitating premature senescence characterized by a substantial increase in the percentage of cells positive for senescence‐associated β‐galactosidase to 15.6% at passage six. Conversely, restricting oxygen tension to physiological hypoxia at 3% oxygen mitigates this metabolic burden, decreasing the senescent population to a mere 0.6% and restoring bioenergetic homeostasis [59]. In addition to delaying senescence, severe hypoxia rapidly activates transcription. Brief exposure of AD‐MSCs to 0.5% oxygen for 48 h prematurely promotes chondrogenic commitment in specific subpopulations by activating the p38 MAPK phosphorylation cascade. This microenvironmental change increases the expression of SOX9, ACAN, and COL2A1, effectively bypassing the need for prolonged treatment with exogenous morphogens [65].

### 2.4 Primary Biological Effectors Following AD‐MSC Isolation

AD‐MSCs possess substantial multipotent differentiation potential; namely, they can differentiate into multiple cell types [66]. However, the contemporary consensus has revised this traditional view. The therapeutic efficacy in vivo rarely relies on direct structural integration or tissue engraftment. Instead, AD‐MSC‐mediated tissue repair is predominantly driven by paracrine immunomodulatory activity. AD‐MSCs modulate immune responses through cytokine and EV secretion, reshaping the functional phenotypes of T cells and macrophages. AD‐MSC‐derived EVs suppress the secretion of proinflammatory cytokines while promoting the release of anti‐inflammatory factors [67, 68]. Interestingly, cellular crosstalk is bidirectional. Inflamed microenvironments do not simply impair MSC function; instead, they actually prime their immunoregulatory potency. For instance, IL‐1β activation increases the ability of AD‐MSCs to promote macrophage polarization toward an anti‐inflammatory phenotype [69]. In bone regeneration and cartilage repair applications, AD‐MSCs secrete multiple cytokines and growth factors to stimulate target cell proliferation and functional recovery [70, 71]. As reported by Abu‐Shahba et al., pathological conditions such as diabetes significantly alter AD‐MSC bioactivity, upregulate proinflammatory factors and downregulate anti‐inflammatory factors [72].

## 3 AD‐MSC‐Derived Extracellular Vesicles (AD‐MSC‐EVs)

Prior to advancing cell‐free therapies, researchers must establish clear definitions for secreted biological effectors. Historically, the regenerative medicine literature has frequently conflated EVs, exosomes, and small extracellular vesicles (sEVs). This inconsistent nomenclature created widespread confusion. By rigorous biological definition, EVs represent a broad category encompassing all nonreplicating, lipid bilayer‐delimited particles. Exosomes constitute a biogenesis‐specific subset that originates exclusively from the endosomal pathway via the exocytosis of multivesicular bodies. Conversely, sEVs are merely physical fractions measuring less than 200 nm, regardless of their biogenetic origin. Because contemporary scalable isolation methods segregate particles based on physical dimensions rather than intracellular biogenesis, they invariably coisolate plasma membrane‐derived ectosomes alongside identically sized endosome‐derived exosomes. Therefore, using the word “exosome” without definitive live‐cell tracing is technically inaccurate. Consistent with the updated Minimal Information for Studies of Extracellular Vesicles (MISEV2023) guidelines, this review universally adopts the comprehensive designation of EVs [18]. This standard term provides a reliable framework for these nanoscale carriers. Ultimately, EVs deliver the bioactive cargo of AD‐MSCs without the physical entrapment and immune rejection risks that whole, living cells face [73].

### 3.1 Core Mechanisms: Epigenetic Silencing and Metabolic Rescue

Operating as epigenetic and metabolic effectors, AD‐MSC‐EVs retain the immunomodulatory signatures of their native perivascular niche. Mechanistically, the functions of specific EV cargos dictate posttranscriptional epigenetic silencing; encapsulated microRNAs explicitly bind the 3′ untranslated regions of target proinflammatory transcripts. The degradation of these transcripts regulates immunometabolism, forcing macrophage repolarization toward tissue‐reparative phenotypes across pathologies, including acute lung injury and diabetic wound healing [74]. Concurrently, within metabolically exhausted microenvironments, AD‐MSC‐EVs mediate bioenergetic restoration via horizontal mitochondrial transfer. Shuttling intact respiratory chain complexes into damaged recipient cells rapidly restores oxidative phosphorylation (OXPHOS) and neutralizes reactive oxygen species, abrogating depolarization‐induced ferroptosis [75, 76]. The therapeutic efficacy of AD‐MSC‐EVs is highly versatile: hypoxic preconditioning accelerates AD‐MSC‐EV‐mediated lymphangiogenesis by stabilizing the hypoxia‐inducible factor‐1α (HIF‐1α) axis and facilitating a metabolic shift toward aerobic glycolysis [77], whereas specialized EVs can induce chondrogenesis and abrogate chondrofibrosis [78].

### 3.2 Cargo Engineering and Targeted Modifications

By penetrating biological barriers and circumventing the poor engraftment and immune rejection limitations associated with cell‐based therapies, AD‐MSC‐EVs show potential across diverse preclinical indications, including wound healing, retinal neuroprotection, nerve regeneration, and myocardial repair [79, 80, 81, 82, 83, 84, 85, 86]. As natural nanocarriers, AD‐MSC‐EVs possess intrinsic targeted delivery capabilities for precision therapy [84, 87, 88]. This engineering strategy selectively reconfigures intraluminal cargoes and extravesicular surface properties. Researchers have used CRISPR to load specific amounts of therapeutic microRNAs. For instance, the overexpression of miR‐125b‐5p and miR‐145a‐5p silences profibrotic signaling in target tissues, facilitating high‐quality tissue regeneration [89, 90]. Extrinsic surface modifications further prevent aberrant systemic clearance. Covalent conjugation of the EV phospholipid bilayer with tissue‐homing peptides, notably the fibrin‐targeting CREKA sequence, promotes the specific accumulation of EVs at lesion sites. This rational surface engineering strategy prolongs local EV retention, combats systemic dissipation, and sustains the elevated local concentrations required for effective tissue repair [91].

### 3.3 The Hemocompatibility Risk: Tissue Factor and Thrombosis

Despite these exciting laboratory results, blood compatibility represents a major safety risk that currently hinders systemic clinical trials. Because EV membranes retain the molecular composition of their parent cells, AD‐MSC‐EVs naturally carry tissue factor (TF/CD142) from their perivascular origins [12, 92]. An intravenous infusion of a high dose of unmodified TF‐bearing EVs initiates the extrinsic coagulation cascade via the factor VII/VIIa complex, precipitating intravascular coagulation and lethal pulmonary thromboembolism [93]. Overcoming this fatal bottleneck requires researchers to abandon oversimplified safety assumptions that erroneously treat EVs as hematologically inert. Rigorous clinical deployment requires the integration of functional hemocompatibility assays into cGMP workflows. Specifically, establishing quantitative batch release criteria based on a calibrated automated thrombogram (CAT) is imperative for a strict evaluation of thrombin generation kinetics prior to clinical administration. Furthermore, resolving these thromboembolic risks necessitates multimodal prevention strategies across the biomanufacturing process chain. These strategies include targeted TF depletion via CRISPR‐Cas9‐mediated F3 gene knockout, heparin conjugation on the surface to render vesicles resistant to thrombosis, and the use of size‐exclusion chromatography (SEC) to systematically fractionate and deplete high‐TF‐expressing vesicular subpopulations.

### 3.4 Industrialization Bottlenecks: Yield Versus Purity

While early clinical trials have validated the safety and feasibility of EVs [94], translational hurdles remain regarding their standardized mass production, control of purity, and targeted delivery in vivo [95]. Traditional differential ultracentrifugation generates intense hydrostatic shear stress, inducing irreversible particle aggregation that masks inherent subpopulation heterogeneity and restricts manufacturing throughput. Tangential flow filtration (TFF) represents a rapid, continuous alternative. However, TFF often accidentally copurifies nonvesicular apolipoproteins, making the precise determination of what the final drug contains difficult. Accordingly, integrating closed 3D bioreactor perfusion systems with high‐resolution orthogonal purification to synergize TFF for rapid concentration with SEC for precise enrichment has become a core cGMP‐compliant manufacturing workflow [96]. This automated processing platform eliminates soluble impurities while retaining the full mechanobiological functionality of EVs [97]. Crucially, this orthogonal purification achieves accurate fractionation of different subpopulations. By stratifying ESCRT‐dependent immunomodulatory subsets from ESCRT‐independent proangiogenic subsets based on specific molecular cargoes, this integrated strategy balances yield and purity trade‐offs, establishing a regulatory prerequisite for tailoring functionally homogenous precision biologics [98].

## 4 Applications of AD‐MSCS With Different Phenotypes in Different Disease Models

Early clinical trials generally treated AD‐MSCs as a uniform therapeutic product and ignored their intrinsic cellular heterogeneity. Current regenerative medicine research advocates for phenotype applications, such that distinct AD‐MSC subsets, including those with immunomodulatory MSC2, proangiogenic, and antifibrotic phenotypes, should be selectively deployed to reverse microenvironment‐specific homeostatic disorders in diseased tissues. Rather than relying on naive structural engraftment, phenotype applications leverage the intrinsic or ex vivo‐primed transcriptomic signatures of AD‐MSCs and their derived EVs to target defined pathological mechanisms.

### 4.1 Preclinical and Clinical Administration Routes: Conserved Rules and Translational Divergence

Comparisons of preclinical models (Table 3) and clinical trials (Table 4) reveal a consistent principle governing cell delivery: anatomical features determine the optimal administration routes. Localized structural defects, including osteochondral lesions, complex fistulas, and regional neurocutaneous injuries, necessitate local injection in both animal models and human patients. This targeted approach bypasses systemic clearance and precisely establishes paracrine signaling gradients at injured sites. Diffuse parenchymal pathologies and advanced systemic autoimmune disease, conversely, can be treated by intravenous injection. This systemic route utilizes the physiological pulmonary first‐pass effect, converting a pharmacokinetic constraint into a highly efficient intra‐alveolar delivery mechanism to shape rapid, body‐wide immunometabolic reprogramming.

**TABLE 3:** Recent advances in research on the treatment of various diseases with AD‐MSCs.

Disease	Frequency	Route	Dose	Auxiliary drugs or materials	Prognosis/outcome	Pathway and mechanism of action	Evidence grading	Statistical comparison	Refs.
Articular cartilage injury	once	Intralesional	4 × 10^5^ AD‐MSCs and 20 mg of the microcarriers	Cartilage‐derived decellularized ECM (cECM); GelMA hydrogel	Achieved excellent cartilage regeneration, inhibited ECM degradation, and integrated well with the surrounding normal tissues.	Dictates fibrosis‐suppressing pathways by mediating paracrine chondroprotective effects; downregulates MMP1, MMP9, MMP13, and ADAMTS4/5 and abrogates proinflammatory IL‐1β signaling.	Level V Preclinical animal experiments (rats)	MMP1, MMP13, ADAMTS4, ADAMTS5 mRNA expression in ADSCs‐MT vs. the control group: *p* < 0.05 or *p* < 0.01.	[99]
Bone defect	once	Intralesional	40 µL of EVs	DMPE‐PEG‐CREKA fibrin‐targeting peptide	Accelerated bone regeneration with a dense trabecular structure, significantly decreased the defect size, and promoted angiogenesis.	Leverages the function of targeted EV cargo via CREKA–fibrin binding to locally trigger angiogenic signaling (upregulating CD31, vWF, and VEGF expression) and dictating M1‐to‐M2 macrophage repolarization alongside osteogenic commitment.	Level V Preclinical animal experiments (rats)	CD31‐positive area vs. the control group: *p* < 0.01; CD31‐positive area vs. the EV group: *p* < 0.01; vWF and VEGF mRNA expression vs. the control group: *p* < 0.05.	[91]
Bone defect	once	Intralesional	2 × 10^4^ AD‐MSCs	Platelet‐rich plasma (PRP) gel, thrombin, calcium chloride, 3D‐printed porous titanium alloy implant (PTI)	Effectively accelerated bone regeneration, promoted vascular network formation, and increased osteogenesis‐related protein expression.	Activates angiogenesis signaling cascades through the sustained release of PDGF, IGF‐1, bFGF, and VEGF, catalyzing the downstream HIF‐1α/SDF‐1α axis while concomitantly upregulating osteogenic transcripts (Runx2, ALP, and OCN).	Level V Preclinical animal experiments (rabbits)	Runx2 fluorescence intensity vs. the control group: *p* < 0.05. HIF‐1α, SDF‐1α, Runx2, ALP, and OCN expression vs. the control group: *p* < 0.05.	[100]
DFU	3 consecutive days	Intralesional	200 µg of EVs	None	Accelerated wound closure, reduced oxidative stress, and promoted angiogenesis.	Governs mitochondrial restoration and bioenergetic rescue by activating the SIRT3/SOD2 pathway; neutralizes catastrophic ROS overload and regulates immunometabolism.	Level V Preclinical animal experiments (mice)	Wound‐healing rate vs. the control: *p* < 0.05. SOD levels vs. the control group: *p* < 0.01. VEGF, VCAM, IL‐1, IL‐6, TNF‐α, and MCP‐1 mRNA expression vs. the control group: *p* < 0.01.	[101]
DFU	once	Intralesional	100 µL of AD‐MSCs	None	Promoted angiogenesis and accelerated wound healing via FGFR2.	Drives localized angiogenic signaling networks by upregulating FGFR2, explicitly catalyzing the downstream FGF, PI3K/Akt, HIF‐1α, VEGF cascade to direct endothelial tubulogenesis and migration.	Level V Preclinical animal experiments (mice)	Wound closure rate vs. the control group: *p* < 0.05. VEGF content vs. the control group: *p* < 0.05. FGFR2, PI3K, AKT, HIF‐1α, and VEGF mRNA expression vs. the control group: *p* < 0.05.	[102]
DFU	once	Intralesional	5 × 10^5^ cells	None	Accelerated wound healing by promoting M2 macrophage polarization.	Executes precise immunometabolic regulation by epigenetically downregulating *EREG* and *CSTA* expression, strategically forcing repolarization from the proinflammatory M1 phenotype to tissue‐reparative M2 macrophages.	Level V Preclinical animal experiments (mice)	Wound closure rate vs. the control group: *p* < 0.01. *EREG* and *CSTA* expression vs. the control group: *p* < 0.01. Expression of M1/M2 markers vs. the control group: *p* < 0.05.	[103]
DFU	Seven times/day	Intralesional	100 µL per mouse (25 µL at 4 points, 1 × 10^10^ particles/mL)	None	Accelerated wound healing, reduced inflammation, and restored mitochondrial function via SIRT1.	Restores bioenergetic allostasis via SIRT1 activation, coordinating mitochondrial restoration and autophagic flux; suppresses oxidative stress to dictate M2‐directed immunometabolic regulation.	Level V Preclinical animal experiments (mice)	Wound‐healing rate vs. the control group: *p* < 0.05. SIRT1 protein levels vs. the control group: *p* < 0.01.	[104]
Diabetic wounds	Three times/day	Intralesional	100 µg of EVs (in 100 µL PBS)	Human acellular amniotic membrane (hAAM) scaffold	Accelerated wound healing, stimulated vascularization, and promoted ECM production.	Leverages the function of EV cargo within hAAM scaffolds to stimulate angiogenic signaling and orchestrate ECM architectural remodeling for rapid wound closure.	Level V Preclinical animal experiments (mice)	Wound closure rate and percentage of M2 macrophages vs. the control group: *p* < 0.05.	[105]
DFU	Once every 3 days	Intralesional	100 µL per mouse (100 µg/mL EVs)	Extracellular matrix (ECM) hydrogel	Reduced inflammation, promoted angiogenesis and collagen deposition, and accelerated wound healing.	Utilizes the sustained function of EV cargo within ECM hydrogels to activate the PI3K/AKT and Wnt/β‐catenin pathways, driving cell migration while regulating immunometabolism via the abrogation of proinflammatory cytokine production.	Level V Preclinical animal experiments (mice)	Wound closure rate vs. the control group: *p* < 0.001. IL‐6, TNF‐α expression vs. the control group: *p* < 0.05.	[106]
Diabetic wounds	once	Intralesional	200 µg of EVs	None	Promoted skin wound healing by regulating epidermal autophagy and rapid re‐epithelialization.	Leverages the function of EV cargo to upregulate the expression of specific genes (NAMPT, CD46, and VAMP3/7) and counter the high‐glucose‐induced impairment.	Level V Preclinical animal experiments (mice)	Wound closure percentage vs. the control group: *p* < 0.05. NAMPT, CD46, VAMP7, and VAMP3 mRNA expression vs. the control group: *p* < 0.001.	[107]
TBI	Once	Intralesional	1 × 10^6^ cells	None	Promoted neural functional recovery, reduced neuronal apoptosis, and inhibited glial cell activation.	Dictates immunometabolic regulation within the neural niche by repolarizing microglia to the M2 phenotype (upregulating Arg‐1) while silencing the expression of M1‐associated proinflammatory mediators (IL‐6 and TNF‐α).	Level V Preclinical animal experiments (rats)	IL‐6, Arg‐1 and TNF‐α vs. the control group: *p* < 0.05.	[108]
TBI	Once	Intralesional	EVs: 20 µg of total protein (2.0 × 10^10^ particles/mL) in 20 µL; AD‐MSCs: 5.0 × 10^5^ cells in 20 µL	None	Facilitated sensorimotor functional recovery, suppressed neuroinflammation, reduced neuronal apoptosis, and promoted neurogenesis.	Leverages the function of targeted EV cargo to specifically penetrate microglia/macrophages, modulating immunometabolic regulation by executing posttranscriptional epigenetic silencing of the classical NF‐κB and p38 MAPK signaling cascades.	Level V Preclinical animal experiments (rats)	MCP‐1, TNF‐α, IL‐1β, and IL‐6 mRNA expression vs. the control group: *p* < 0.05. IL‐1β and IL‐6 levels vs. the control group: *p* < 0.05.	[109]
SCI	Once	Intralesional	∼10^4^ cells	Fibrin matrix hydrogel	Improved cell retention, survival, and differentiation; reduced cavitation and astrogliosis. However, the original locomotor functions were not fully regained.	Employs fibrin matrix compartmentalization to structurally support survival and differentiation, executing local immunometabolic regulation and suppressing fibrotic suppression to attenuate astrogliosis.	Level V Preclinical animal experiments (rats)	MBP and OSP mRNA expression vs. the control group: *p* < 0.05.	[110]
LSCD	Once	Intralesional	10^6^ cells/mL seeded on a 4 × 4 cm amniotic membrane	Topical levofloxacin (0.5%) and betamethasone (0.1%) applied twice daily	Improved corneal transparency, decreased neovascularization and inflammation, and reconstructed the epithelial structure.	Modulates the ocular microenvironment by suppressing fibrotic pathways and blocking inflammation through decreases in the levels of proangiogenic factors (VEGF and MMP‐2) and an increase in the level of the anti‐inflammatory cytokine IL‐10.	Level V Preclinical animal experiments (rats)	VEGF‐A, MMP‐2, MCP‐1, IL‐6, TNF‐α, TGF‐β, and IL‐10 mRNA expression vs. the control group: *p* < 0.05.	[111]
Corneal decompensation	Once	Intralesional (followed by electroporation)	4.5 × 10^8^ or 2.25 × 10^8^ EVs in 10 µL	None	Promoted wound healing, increased the corneal endothelial cell density, decreased opacity, and reduced senescence and autophagy.	Deploys the function of EV cargo to antagonize TGF‐β‐ and oxidative stress‐mediated senescence and the endothelial–mesenchymal transition (EndoMT), suppressing fibrotic pathways via the Hippo signaling axis (upregulating pYAP).	Level V Preclinical animal experiments (rats)	In vitro cell viability and number of Ki67^+^ cells vs. treatment with TGF‐β/H_2_O_2_: *p* < 0.05. N‐cadherin/GAPDH and pYAP/YAP ratios vs. treatment with TGF‐β/H2O2: *p* < 0.05.	[112]
LSCD	Once	Intralesional	2.5 × 10^5^ cells seeded onto 2 × 2 cm pieces of amniotic membrane	Topical Maxitrol and tobramycin, systemic buprenorphine and enrofloxacin, and a transeptal triamcinolone injection into the amniotic membrane	Restrained the evolution of corneal neovascularization and opacity, reduced inflammation, and partially restored corneal and limbal epithelial phenotypes.	Acts via paracrine signaling pathways to suppress pathological angiogenesis and facilitate the recovery of host corneal and limbal epithelial phenotypes (CK3, p63, CK15, and E‐cadherin expression).	Level V Preclinical animal experiments (rabbits)	Neovessel length vs. the control group: *p* < 0.05. Percentage of p63‐positive cells vs. the control group: *p* < 0.001.	[113]
KCS	1 time/d for 8 days	Intralesional	1 × 10^5^ cells per eye	None	Accelerated corneal healing, promoted transient membrane gland repair, increased tear secretion, and suppressed periocular inflammation.	SPARC overexpression hyperactivates the PI3K/Akt and ERK/MAPK cascades, suppressing fibrotic pathways by downregulating proinflammatory (IL‐6, IL‐1β, and TNF‐α) and matrix‐degrading (MMP‐9) proteins and executing immunometabolic regulation.	Level V Preclinical animal experiments (dogs)	Serum IL‐6, IL‐1β, and TNF‐α levels vs. the control group: *p* < 0.01. Cell viability and IL‐6, TNF‐α, and MMP‐9 mRNA expression vs. the control group: *p* < 0.01.	[114]
ILD	once	i.v.	1.0 × 10^5^ cells	Low‐molecular‐weight heparin (LMWH) was used in vitro for AD‐MSC preprocessing	Significantly reduced lung fibrosis and collagen deposition.	Suppresses fibrotic pathways by activating the SDF‐1/CXCR‐7 axis to increase HGF secretion, effectively abrogating diffuse pulmonary fibrogenesis and the systemic release of inflammatory cytokines.	Level V Preclinical animal experiments (mice)	Fibrotic area, fibrosis score, and hydroxyproline content vs. the control group: *p* < 0.05. Relative mRNA expression of MMP‐9, TIMP‐1, TNF‐α, IL‐6, IL‐1β, IDO‐1, and IL‐1RA vs. the control group: *p* < 0.05.	[115]
IPF	once	i.v.	1.5 × 10^6^ cells	None	Improved the pathogenesis of pulmonary fibrosis and decreased the collagen content.	Suppresses fibrotic pathways by upregulating *PTPRR*, which aggressively dephosphorylates and blocks the ERK1/2 signaling cascade, strictly inhibiting the transcription of *ACTA2* (α‐SMA) and aberrant collagens.	Level V Preclinical animal experiments (mice)	Ashcroft score and collagen content vs. the control group: *p* < 0.05. *ACTA2*, Col1A2, and Col3A1 mRNA expression vs. the control group: *p* < 0.05.	[116]
COPD	once	i.v.	1.0 × 10^6^ cells	None	Ameliorated emphysema histologically and improved pulmonary function.	Facilitates alveolar repair and suppresses fibrosis through the mesenchymal‐to‐epithelial transition (MET), promoting the direct differentiation of these cells into functional alveolar epithelial cells to reinstate structural integrity.	Level V Preclinical animal experiments (mice)	Mean linear intercept (Lm) and FEV0.1 vs. the model group: *p* < 0.01.	[117]
ARDS	once	i.v.	2.0 × 10^5^ cells	None	Attenuated neutrophil infiltration, decreased lung injury scores, and reduced interstitial fibrosis.	Executes acute immunometabolic regulation by strictly inhibiting localized neutrophil recruitment and infiltration and neutralizing myeloperoxidase (MPO) levels to abrogate early acute lung injury.	Level V Preclinical animal experiments (mice)	Lung injury score vs. the control group on Day 2: *p* < 0.05. MPO levels vs. the control group on Day 7: *p* < 0.01.	[118]
Hypospadias and urethral stricture	once	Intralesional	50% (v/v) EVs blended in the core layer solution of the nanoyarn scaffold	None	Urethral repair did not lead to either signs of urethral stricture or scar formation at 4 weeks postsurgery; a multilayer epithelium formed.	Harnesses the function of EV cargo to drive immunometabolic regulation (CD206^+^ M2 polarization), explicitly inhibit excessive Col 1A1 deposition by suppressing fibrotic pathways, and stimulate angiogenic signaling in CD31^+^ cells.	Level V Preclinical animal experiments (Rabbits)	Immunofluorescence staining for AE1/AE3, CD31, and CD206 vs. the control group: *p* < 0.01. COL I expression vs. the control group: *p* < 0.05.	[119]
Urethral stricture	once	Intralesional	Scaffold: 5 × 10^3^ cells/mL. Suture: 1 × 10^6^ cells/mL	Sildenafil at 2 mg/kg daily via oral gavage for 4 weeks	**Substantial** restoration of the urethral caliber with no residual narrowing at 10 weeks after surgery; regeneration of the epithelium and smooth muscle.	Coordinates the suppression of fibrotic pathways by downregulating the expression of the mediator MMP2; the synergistic effect of sildenafil modulates antifibrotic and angiogenic signaling and activates progenitors (CD117^+^).	Level V Preclinical animal experiments (rabbits)	Histopathological grading (fibrosis, angiogenesis, inflammatory cell infiltration, collagenization) among experimental groups: *p* < 0.05. Immunoreactivity for Uroplakin, Desmocollin, CD117, and MMP2 vs. the control group: *p* < 0.05.	[120]

Abbreviations: ARDS, acute respiratory distress syndrome; AVN, avascular necrosis; COPD, chronic obstructive pulmonary disease; DFU, diabetic foot ulcer; ILD, interstitial lung disease; IPF, idiopathic pulmonary fibrosis; i.v., intravenous; KCS, keratoconjunctivitis sicca; LSCD, limbal stem cell deficiency; SCI, spinal cord injury; TBI, traumatic brain injury.

Note on evidence grading: The evidentiary strength of the cited studies was objectively stratified according to trial phase and study design, adapted from the Oxford Centre for Evidence‐Based Medicine (OCEBM) levels of evidence. Level I: High‐quality, adequately powered randomized controlled trials (RCTs) or systematic reviews/meta‐analyses of RCTs. Level II: Lesser‐quality RCTs, prospective cohort studies, or systematic reviews of these studies. Level III: Retrospective cohort studies or case–control studies. Level IV: Case series, single‐arm observational studies, historically controlled studies, or uncontrolled Phase I/IIa safety and feasibility trials. Level V: Preclinical in vivo animal models, in vitro bench research, expert opinions, or single‐case reports.

**TABLE 4:** Recent advances in clinical trials utilizing AD‐MSCs for the treatment of multiple diseases.

Disease	Source	Details	Route	Dose	Frequency	Nation	NCT	Phase	Outcomes	Adverse events (AEs)	Statistical comparison and evidence grading	Refs.
IC	Autologous adipose tissue	AD‐MSCs‐SVF	Intralesional	30–60 million cells	once	Mexico, India	NCT01502514	I	Improved LVEF and increased ventricular wall thickness.	No serious adverse events directly related to the cell therapy occurred.	LVEF was increased at 3 and 6 months (*p* < 0.01). The 6MWT distance was improved at 3 months (*p* < 0.01). Wall thickness was improved at 3, 6, and 12 months (*p* < 0.05). Level IV	[121]
Psoriasis	Allogeneic adipose tissue	AD‐MSCs	i.v.	0.5 × 10^6^ cells/kg	3 times	China	NCT03265613	Pilot Study	No significant difference in treatment efficacy.	No serious adverse events directly related to the cell infusion occurred.	No statistically significant differences in PASI, BSA, VAS, or DLQI scores from the baseline values. Level IV	[122]
SCI	Autologous adipose tissue	AD‐MSCs	Intralesional	1 × 10^8^ cells	once	USA	NCT03308565	I	Spinal cord injury severity improved in seven of 10 patients.	No serious adverse events occurred.	No *p* values as it was a single‐arm study. Level IV	[123]
RA	Autologous adipose tissue	AD‐MSCs	i.v.	2 × 10^8^ cells	once	USA	NCT03691909	I/IIa	Improvements in joint swelling and tenderness.	Four mild AEs were classified as treatment related.	Swollen and tender joint counts were reduced at Week 52 (*p* < 0.013). Level IV	[124]
AGA	Allogeneic adipose tissue	AD‐MSC‐CM	Intralesional	2 mL with 5% minoxidil	3 times	Indonesia	NCT05296863	Pilot Study	No significant difference between two groups.	Most cases involved mild local irritation, itching, and folliculitis caused by minoxidil. One case of contact dermatitis occurred, with no serious adverse reactions reported.	HC, HD, TR, VR, and MT improved in the treatment group at Week 6 (*p* < 0.05). Level II	[125]
CS	Autologous adipose tissue	AD‐MSCs	Intralesional	1 × 10^8^ cells	once	Taiwan, China	NCT02813512	I	Six‐month follow‐up revealed the improvement in treatment outcomes.	No treatment‐related serious adverse events occurred.	No *p* values because it was a single‐arm trial. Level IV	[126]
RCD	Autologous adipose tissue	AD‐MSCs	Intralesional	1.0 × 10^7^, 5.0 × 10^7^, or 1.0 × 10^8^ cells	once	Korea	NCT02474342	I/IIa	The high‐dose group showed a significant improvement in common shoulder joint function scores within 2 years.	No treatment‐related serious adverse events occurred.	SPADI scores were decreased in the low‐, mid‐, and high‐dose groups (*p* < 0.05). The volume of bursal‐sided defects in the high‐dose group was decreased (*p* < 0.05). Level III	[127]
SD	Autologous adipose tissue	AD‐MSCs	i.v.	1 × 10^6^ cells/kg	once	Vietnam	NCT03346967	I/IIa	Patients experienced an improved sexual quality of life, with male patients showing a significant increase in testosterone levels that persisted for up to 6 months.	Two cases were considered intervention‐related: surgical site infection and elevated liver enzyme levels.	In males, testosterone levels increased (*p* < 0.05). Significant improvements in sexual quality of life were observed (*p* < 0.05). Level IV	[128]
T1D	Allogeneic adipose tissue	AD‐MSCs	i.v.	1 × 10^6^ cells/kg with 2000 IU/d cholecalciferol	AD‐MSCs were administered once; cholecalciferol was administered for 3–6 months	Brazil	NCT03920397	II	Potential clinical benefits with lower exogenous insulin requirements were observed in the intervention group.	One case of complete recovery following central retinal vein occlusion.	The intervention group had a lower insulin requirement (*p* = 0.01) and HbA1c level (*p* = 0.03). Level II	[129, 130]
CKD	Allogeneic adipose tissue	AD‐MSCs	i.v.	6.4 × 10^7^, 19.2 × 10^7^, or 32.0 × 10^7^ cells	once	Taiwan, China	NCT02933827	I/II	The treatment was well tolerated, with a significant reduction in the risk of deterioration. The low‐dose group exhibited the best short‐term stabilization of renal function.	Two cases of slowed speech and mental dullness occurred in the high‐dose group; no fatal adverse events were reported.	The group receiving AD‐MSC therapy had a lower risk of eGFR decline (*p* < 0.05). Level III	[131, 132]
RFU	Allogeneic adipose tissue	AD‐MSC‐PLCs	Intralesional	1.5 × 10^8^ cells/cm^2^	3 times	Japan	jRCTa030200053	I/IIa	Ulcer areas were decreased, with most patients achieving epithelial healing.	A total of 9 mild/moderate adverse events were reported, with no serious adverse events.	No *p* values as it was a single‐arm study. Level IV	[133]
RFU	Allogeneic adipose tissue	AD‐MSCs	Intralesional	6 × 10^6^ cells	once	Turkey	NA	I/II	The mean time to wound closure was significantly shorter in the intervention group.	No treatment‐related serious adverse events occurred.	The mean time to wound closure was shorter in the AD‐MSC group (*p* = 0.002) Level II	[134]
AIS	Allogeneic adipose tissue	AD‐MSCs	i.v.	1 × 10^6^ cells/kg	once	Spain	EudraCT: 2011‐003551‐18; NCT01678534	IIa	No significant difference in treatment outcomes between groups.	No treatment‐related serious adverse events occurred.	No significant differences between the AD‐MSC and placebo groups. Level II	[135]
ARDS	Allogeneic adipose tissue	AD‐MSCs	i.v.	1 × 10^6^ cells/kg	once	China	NCT01902082	I	Overall safety and feasibility were demonstrated. Serum SP‐D levels in the treatment group decreased significantly by Day 5.	No treatment‐related serious adverse events occurred.	Serum SP‐D levels on Day 5 were lower vs. baseline. (*p* = 0.027). Level II	[136]
ARVD	Autologous adipose tissue	AD‐MSCs	Intralesional	1 × 10^5^, 2.5 × 10^5^, or 5.0 × 10^5^ cells/kg	once	USA	NCT02266394	Ia	Improvements in tissue ischemia and hypoxia. The high‐dose group showed a significant increase in the GFR.	NA	Stenotic kidney renal blood flow increased (*p* = 0.007) and fractional hypoxia decreased (*p* = 0.01). High‐dose group experienced the increase in GFR (*p* = 0.045). Level III	[137]
CD	Allogeneic adipose tissue	AD‐MSCs	Intralesional	120 million cells	once	Europe and Israel	NCT01541579	III	The proportion of symptom relief in the experimental group was significantly higher than that in the placebo group.	In the experimental group, 17% of patients experienced treatment‐related adverse events, most commonly anal abscesses and rectal pain, which are inherent to perianal fistulas in patients with Crohn's disease and for which a causal relationship with the cell therapy remains uncertain.	Combined remission was significantly increased (*p* = 0.024). Level II	[10]
CD	Autologous adipose tissue	AD‐MSCs	Intralesional	18–104 mL	One to three times	Denmark	NCT03803917	Not a formal clinical trial	Six months after the last injection, 57% of patients achieved symptom remission, with an overall treatment response rate of 76%.	No treatment‐related serious adverse events occurred.	No *p* values as it was a single‐arm study. Level IV	[138]
LBP	Autologous adipose tissue	AD‐MSCs with hyaluronic acid (HA)	Intralesional	6.0 × 10^6^ cells with HA	once	Korea	NCT05011474	I	Six of eight patients achieved symptomatic relief; four patients experienced a radiographic improvement.	None.	VAS scores at 3 months (*p* = 0.0181) and 6 months (*p* = 0.0325) compared with the baseline value. Level IV	[139]
SPMS	Autologous adipose tissue	AD‐MSCs	i.v.	1 × 10^6^ or 4 × 10^6^ cells/kg	once	Spain	NCT01056471	I/II	No statistically significant differences were observed between the treatment group and the placebo group in terms of EDSS scores and the number of lesions detected using MRI.	No treatment‐related serious adverse events occurred.	No significant differences. Level II	[140]
SMA	Allogeneic adipose tissue	AD‐MSCs	Intralesional	1 × 10^6^, 2 × 10^6^, or 5 × 10^6^ cells/kg	A total of 3 times (once every 15 days)	Iran	NCT02855112; IRCT2015073023417N1	I	Patients in the intervention group presented significantly improved tibial nerve motor evoked potential amplitude responses.	No serious adverse events occurred.	Tibial nerve motor amplitude response compared with the control group (*p* = 0.029). Level II	[141]
AMI	Autologous adipose tissue	AD‐MSCs	Coronary artery infusion	∼17.4 ± 4.1 × 10^6^ cells	once	The Netherlands	NCT00442806	I/IIa	The LVEF showed an improving trend, with a reduction in the area of left ventricular myocardial infarction and a significant improvement in myocardial perfusion defects.	A total of 33% of patients in the cell therapy group experienced severe adverse events, but all were confirmed to be unrelated to the cell infusion therapy.	The infarct size was reduced in the AD‐MSC group (*p* = 0.002), and the perfusion defect improved in the AD‐MSC group (*p* = 0.004). Level II	[142]
KOA	Allogeneic adipose tissue	AD‐MSCs	Intra‐articular	100 × 10^6^ cells	once	Iran	IRCT20080728001031N23	II	The treatment group had significantly improved efficacy.	No serious adverse events occurred.	Total WOMAC, VAS, KOOS, and SF‐36 scores compared with the control group (*p* < 0.001). Level II	[143]
KOA	Allogeneic adipose tissue	AD‐MSCs	Intra‐articular	6.7 × 10^6^ or 4.0 × 10^7^ cells	once	Taiwan, China	NCT03943576	I	One‐year follow‐up confirmed the treatment was safe and well tolerated; the experimental group showed improvements in both pain and knee function.	No treatment‐related serious adverse events occurred.	Improvements in WOMAC total, pain, and physical function scores in the high‐dose group compared with the baseline values (*p* < 0.05). Level IV	[144]
KOA	Allogeneic adipose tissue	AD‐MSCs	Intra‐articular	16 million, 32 million, or 64 million cells	once	Taiwan, China	NCT02784964	I/II	The overall safety and tolerability were good. The experimental group exhibited a faster functional improvement and sustained efficacy over a longer period.	No serious adverse events occurred.	Significant difference in the mean WOMAC total scores (*p* = 0.0113). Differences in the VAS pain score and KSCRS functional activities score (*p* < 0.05). Level II	[145]
SLE	Allogeneic adipose tissue	AD‐MSCs	i.v.	2 × 10^6^ cells/kg	once	Iran	IRCT2016090729747N1	I	Preliminary data indicated early reductions in urine protein levels and an observational trend toward disease activity attenuation.	The treatment protocol resulted in no major adverse events.	Urine protein levels were decreased (*p* = 0.008). Median SLEDAI‐2K scores decreased at Month 6 (*p* = 0.007). Level IV	[146]

Abbreviations: AD‐MSC‐CM, AD‐MSC‐conditioned medium; AD‐MSC‐PLCs, adipose‐derived mesenchymal stem cell line‐derived platelet‐like cells; AGA, androgenetic alopecia; AIS, acute ischemic stroke; AMI, acute myocardial infarction; ARDS, acute respiratory distress syndrome; ARVD, atherosclerotic renovascular disease; CD, Crohn's disease; CKD, chronic kidney disease; CS, cerebral stroke; IC, ischemic cardiomyopathy; i.v., intravenous; KOA, knee osteoarthritis; RA, rheumatoid arthritis; RCD, rotator cuff disease; RFU, refractory foot ulcer; SCI, spinal cord injury; SD, sexual dysfunction; SLE, systemic lupus erythematosus; SMA, spinal muscular atrophy; SPMS, secondary progressive multiple sclerosis; T1D, type 1 diabetes.

Note on evidence grading: The evidentiary strength of the cited studies was objectively stratified according to trial phase and study design, adapted from the Oxford Centre for Evidence‐Based Medicine (OCEBM) levels of evidence. Level I: High‐quality, adequately powered randomized controlled trials (RCTs) or systematic reviews/meta‐analyses of RCTs. Level II: Lesser‐quality RCTs, prospective cohort studies, or systematic reviews of these studies. Level III: Retrospective cohort studies or case–control studies. Level IV: Case series, single‐arm observational studies, historically controlled studies, or uncontrolled Phase I/IIa safety and feasibility trials. Level V: Preclinical in vivo animal models, in vitro bench research, expert opinions, or single‐case reports.

However, the current data also reveal a substantial translational disconnect between preclinical efficacy and clinical outcomes. Preclinical studies, which utilize acute injury configurations in metabolically resilient animals, frequently deploy supraphysiological cell doses combined with advanced bioengineered scaffolds to facilitate tissue repair. Clinical trials confront hostile, avascular, and chronically inflamed human tissue microenvironments, leading to distinct, strictly regulated dosing protocols. Trials using systemic intravenous injections in humans strictly enforce weight‐calibrated dosing (typically 1.0–2.0 × 10^6^ cells/kg) to restrict autoreactive T‐cell expansion while preventing microvascular thromboembolism. Localized clinical interventions deploy massive, fixed‐cell aliquots (ranging from 1.0 × 10^7^ to 1.2 × 10^8^ cells) suspended in basic carriers, reflecting stringent regulatory constraints on advanced medicinal products. Because of this harsh environment, unprotected cultured cells often undergo rapid postinjection loss, restricting the overall therapeutic efficacy. Narrowing this translational gap will refine future clinical delivery strategies, requiring the implementation of clinically compliant delivery systems and standardized preconditioning protocols to sustain stable paracrine function under chronic pathological conditions in humans.

### 4.2 Musculoskeletal Regeneration: Chondro‐Osteogenic Microtissue Remodeling

The application of AD‐MSCs in the repair of the musculoskeletal system is progressively shifting toward functionalized microtissue engineering and composite bioscaffolds (Figure 2B). Everts et al. [147] proposed that combining a tissue‐derived stromal vascular fraction (t‐SVF) enriched in AD‐MSCs with high‐density platelet‐rich plasma (HD‐PRP) can promote soft tissue repair. With respect to bone defect repair mechanisms, Wang et al. [100] utilized 3D‐printed porous titanium alloy scaffolds loaded with AD‐MSCs and a PRP gel (PTI/AP). They demonstrated that this system not only provides mechanical support but also significantly upregulates the expression of HIF‐1α and its downstream factor SDF‐1α through sustained growth factor release, driving localized angiogenesis and new bone formation [100]. After binding to the CREKA peptide, AD‐MSC‐EVs can precisely target bone defect sites, prolong their retention, and increase repair efficiency, promoting bone repair in a rat femoral defect model [91].

**FIGURE 2:**
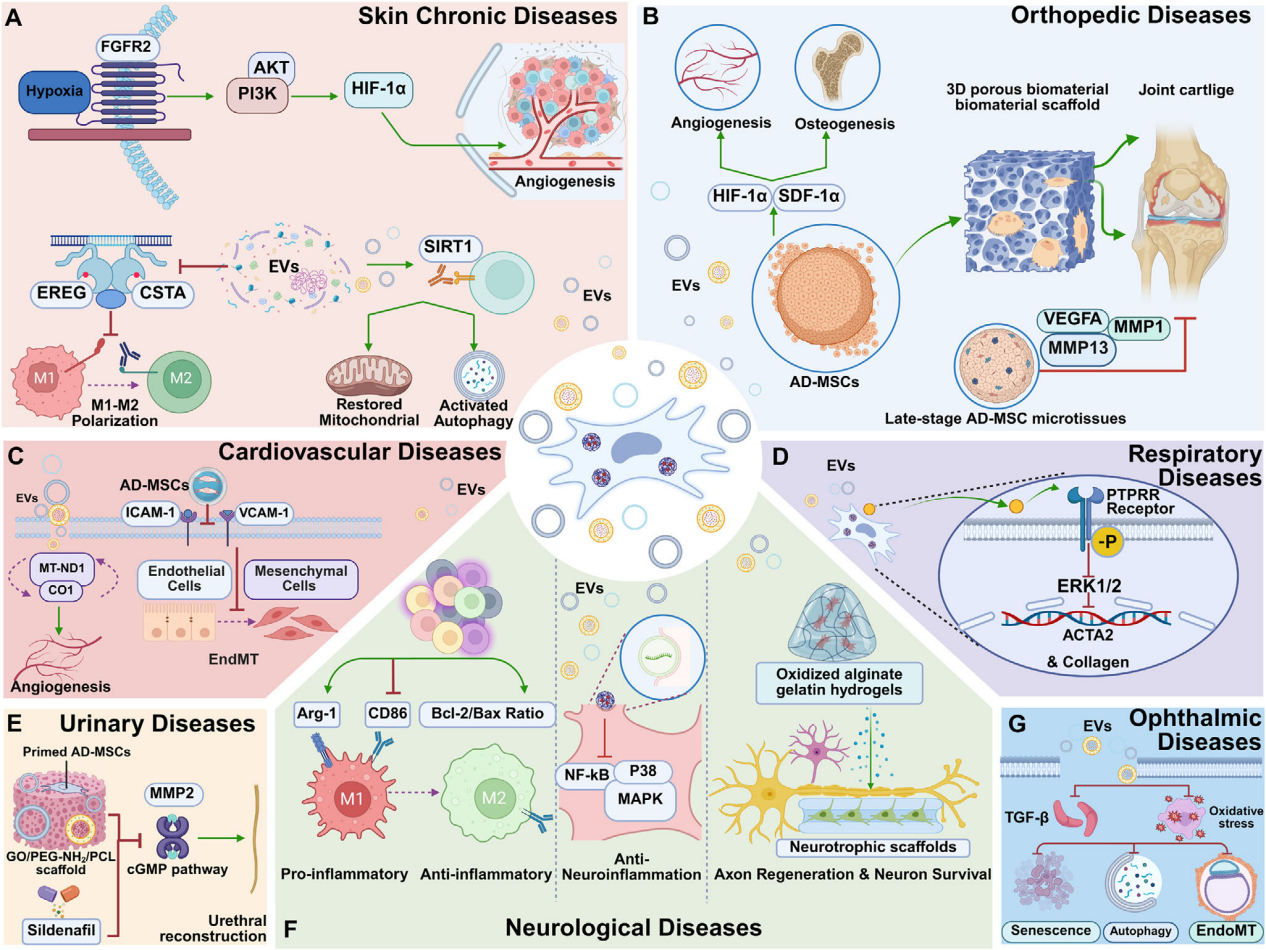
Pleiotropic therapeutic applications and underlying molecular mechanisms of AD‐MSCs and their derived extracellular vesicles (EVs). This schematic comprehensively illustrates the multisystemic regenerative and immunomodulatory pathways governed by AD‐MSC‐based therapies across diverse organ systems. (A) Cutaneous repair: AD‐MSCs and their EVs accelerate diabetic wound healing by promoting angiogenesis via the FGFR2 signaling axis, promoting macrophage polarization toward the reparative M2 phenotype by downregulating *EREG* and *CSTA*, and restoring mitochondrial bioenergetic function via SIRT1. (B) Musculoskeletal regeneration: Functional microtissues and composite bioscaffolds support chondro‐osteogenic remodeling and protect against cartilage matrix degradation by downregulating matrix metalloproteinases (MMP‑1 and MMP‑13) and VEGFA. (C) Cardiovascular diseases: These therapies promote myocardial repair and attenuate atherosclerosis through intercellular mitochondrial transfer (upregulating *MT‐ND1* and *MT‐CO1*) and inhibition of endothelial‑to‑mesenchymal transition (EndMT) via downregulation of ICAM‐1 and VCAM‐1. (D) Pulmonary repair and regeneration: In respiratory disorders, these therapies primarily aim to slow disease progression, reverse pathological matrix deposition, and promote structural tissue repair. In models of idiopathic pulmonary fibrosis and emphysema, the mechanisms include upregulation of *PTPRR*, which inhibits the ERK1/2 cascade and *ACTA2* transcription, thereby reducing excessive fibrosis, as well as promotion of mesenchymal‑to‑epithelial transition (MET) to restore alveolar structure. (E) Urogenital tract reconstruction: To reduce pathological scarring and slow disease progression in the genitourinary tract, therapeutic strategies target aberrant profibrotic pathways to promote tissue‐specific structural recovery. Using complex urethral strictures as a representative clinical indication, one established approach combining AD‑MSCs with functionalized nanoscaffolds (e.g., GO/PEG‐NH_2_/PCL) downregulates profibrotic mediators such as MMP‐2 to attenuate severe tissue scarring, while also supporting regeneration of functional vascular networks and stratified urothelium. (F) Neurological applications: In the central nervous system, AD‐MSCs and their EVs exert neuroprotective effects and promote tissue regeneration. They alleviate neuroinflammation by promoting microglial repolarization toward the reparative M2 phenotype (via Arg‑1 upregulation), inhibiting NF‑κB and p38 MAPK signaling, and regulating neuronal apoptosis via modulation of the Bcl‑2/Bax ratio. In addition to neuroprotective effects, they also support neuroregeneration by promoting differentiation of neural and oligodendrocyte precursors and enhancing axonal remyelination. (G) Ophthalmic applications: Paracrine signaling remodels the ocular microenvironment and preserves the limbal stem cell niche by alleviating oxidative stress and inhibiting TGF‐β‐driven endothelial‑to‑mesenchymal transition (EndoMT). This figure was created at BioRender.com. *ACTA2*, actin alpha 2; α‐SMA, alpha smooth muscle actin; Arg‐1, arginase‐1; Bax, Bcl‐2‐associated X protein; Bcl‐2, B‐cell lymphoma 2; *CSTA*, cystatin A; EndMT, endothelial‐to‐mesenchymal transition; EndoMT, endothelial–mesenchymal transition; *EREG*, epiregulin; ERK1/2, extracellular signal‐regulated kinase 1/2; FGFR2, fibroblast growth factor receptor 2; GO/PEG‐NH_2_/PCL nanoscaffold; ICAM‐1, intercellular adhesion molecule 1; MMP1, matrix metalloproteinase‐1; MMP2, matrix metalloproteinase‐2; MMP13, matrix metalloproteinase‐13; *MT‐CO1*, mitochondrially encoded cytochrome c oxidase 1; *MT‐ND1*, mitochondrially encoded NADH dehydrogenase 1; *PTPRR*, protein tyrosine phosphatase receptor type R; p38 MAPK, p38 mitogen‐activated protein kinase; SIRT1, sirtuin 1; TGF‐β, transforming growth factor beta; VCAM‐1, vascular cell adhesion molecule 1; VEGFA, vascular endothelial growth factor A.

Clinical translation corroborates these preclinical mechanisms. Intra‐articular AD‐MSC administration strongly modulates immunometabolism, attenuating progressive joint degradation in patients with knee osteoarthritis [143, 144, 145]. Spatial bioengineering has similarly achieved success; intradiscal AD‐MSC spheroids limit cellular leakage to promote structural stabilization in individuals with chronic discogenic pain [139]. Direct tendon injection repairs bursal lesions and supports long‐term functional tendon‐muscle recovery [127]. An intravenous infusion of AD‐MSCs into individuals with rheumatoid arthritis confers notable clinical joint amelioration and partially resolves systemic inflammation, indicating a trend toward local paracrine therapeutic effects rather than broad immunosuppression [124].

Current translational evidence suggests that regenerative outcomes were overestimated in early studies. For instance, single‐case clinical observations of patients with Ficat Stage I osteonecrosis of the femoral head (ONFH) treated with core decompression combined with autologous microfragmented adipose tissue (Lipogems) have suggested the possible suppression of necrotic progression [148, 149]. Nevertheless, the evidence from *N* = 1 patient lacks intrinsic statistical validity. These limited preliminary results remain preliminary and hypothesis‐generating. Large‐sample, adequately powered randomized controlled trials (RCTs) are therefore needed to validate clinical efficacy.

Notably, regarding the selection of MSC sources, Costela‐Ruiz et al. [150] reported that compared with AD‐MSCs, BM‐MSCs exhibit superior osteogenic mineralization capacity under standard induction conditions. However, contemporary transcriptomic analyses (RNA‐seq) revealed that AD‐MSC microtissues exhibited significantly downregulated expression of matrix metalloproteinases (MMP13 and MMP1) and vascular endothelial growth factor A (VEGFA) during late differentiation. This property enables these cells to more effectively inhibit cartilage matrix degradation and maintain the stability of the cartilage phenotype in inflammatory microenvironments, suggesting superior inflammatory resistance for treating complex inflammatory osteoarthritis [99, 150].

### 4.3 Cutaneous Repair: Immunometabolic Modulation of the Wound Microenvironment

In the treatment of diabetic foot ulcers (DFUs), burns, and degenerative skin diseases, AD‐MSCs and their EVs facilitate wound healing by activating neoangiogenesis and remodeling disordered immunometabolic profiles within a hostile wound microenvironment (Figure 2A) [101, 134, 151, 152, 153, 154, 155, 156, 157]. Mechanistic research has revealed that AD‐MSCs activate the angiogenic capacity of endothelial cells under hypoxic conditions by upregulating fibroblast growth factor receptor 2 (FGFR2) expression in the ulcer tissue and activating the downstream PI3K/Akt/HIF‐1α signaling axis [102]. In terms of the regulation of the immune microenvironment, AD‐MSCs restrain sustained M1 macrophages in chronic wounds by downregulating epiregulin (*EREG*) and cystatin A (*CSTA*) expression, driving their transition toward the reparative M2 phenotype [103]. AD‐MSC‐EVs also activate autophagy pathways, promoting the functional recovery and regeneration of keratinocytes and thereby accelerating wound epithelialization [107].

Crucially, early clinical translation requires a meticulous evaluation of the evidence. A Phase I/II trial by Uzun et al. [134] demonstrated that a single intralesional injection of allogeneic AD‐MSCs achieved its primary endpoint, establishing the safety and tolerability of this treatment. While exploratory secondary endpoints indicated accelerated mean healing times (31.0 days vs. 54.8 days) and a 90% wound closure rate, interpreting Phase I/II safety data as proof of efficacy is premature. Adequately powered, randomized Phase III trials are needed to substantiate these efficacy signals.

The topical application of cryopreserved platelet‐like cells successfully precipitates refractory ulcer epithelialization without exacerbating local ischemia, overcoming the limited viability of fresh cells [133]. A localized injection of uncultured microfragmented adipose tissue preserves the stromal niche to precipitate refractory fistula healing without ex vivo expansion [138]. In addition to localized repair, a safety profile for the systemic infusion for severe psoriasis has been established; despite failing to induce a short‐term decrease in severity, the intervention has achieved long‐term remission in subsets of patients, indicating sustained therapeutic potential [122]. In patients with androgenetic alopecia, the use of AD‐MSC‐conditioned medium is not significantly superior to standard therapies, indicating the need for advanced delivery vehicles to recapitulate the paracrine activity observed in vitro and achieve definitive clinical efficacy [125].

In addition to wound repair, AD‐MSC‐EVs suppress high glucose‐induced fibroblast senescence, upregulate SIRT1 expression, alleviate oxidative stress, and restore bioenergetic function in damaged macrophages [101, 104, 158, 159]. Clinically, the administration of allogeneic AD‐MSCs or their EVs shortens DFU healing time and reduces the amputation risk [133, 134, 160]. Innovative cell products such as adipose‐derived mesenchymal stem cell line‐derived platelet‐like cells (AD‐MSC‐PLCs) [133] or those that integrate EVs with bioscaffolds have been developed to ensure sustained paracrine release, improve the local retention of the treatment, and combat diabetic wounds [105, 106].

### 4.4 Neurological and Ocular Applications: Microenvironmental Remodeling and Neuroprotection

In both the central nervous system (Figure 2F) and ophthalmic organs (Figure 2G), AD‐MSCs mediate repair predominantly via extensive immunomodulatory remodeling and the suppression of pathological fibrosis. In traumatic brain injury (TBI), Jiang et al. [108] demonstrated that the allogeneic transplantation of canine AD‐MSCs downregulates CD86 expression and upregulates Arg‐1 expression, polarizing microglia from a proinflammatory M1 phenotype to an anti‐inflammatory M2 phenotype. This cascade limits the excessive activation of microglia and astrocytes, abrogating neuroinflammation within the CNS [108]. In addition, EVs can be internalized by microglia via endocytosis, blocking phosphorylation cascades in the NF‐κB and p38 MAPK pathways to alleviate neuroinflammation [109].

Hostile ischemic niches typically accelerate the apoptotic elimination of unprotected cells, making engineered bioscaffolds indispensable for cell survival during spinal cord regeneration [161, 162]. The incorporation of fibrin matrices induces neural and oligodendrocyte precursor differentiation, reducing lesion cavitation upon transplantation [110]. Combined with oxidized alginate gelatin hydrogels, asiatic acid enhances neurotrophic activity and promotes neurite elongation [110, 163], whereas specialized composite scaffolds further support axonal regeneration and remyelination [164]. Additionally, advanced graphene‐based substrates apply mild electrical stimulation to increase neuronal proliferation and calcium signaling, reducing the reliance on conventional chemical induction regimens [165].

In clinical settings, the administration routes determine the therapeutic efficacy of AD‐MSC‐based interventions. Stereotactic intracerebral implantation uniquely bypasses the blood–brain barrier in individuals with chronic stroke. Targeted cell delivery to the peri‐infarct region improves motor functional recovery, with the therapeutic outcomes closely correlated with local neuroimaging signatures along the implantation pathway [126]. Intrathecal delivery ameliorates functional deficits caused by spinal cord injury, although asymptomatic nerve root aggregation necessitates close clinical surveillance [123]. Intrathecal delivery of the clonogenic side population of AD‐MSCs provides potent neurotrophic support to restore motor function in patients with spinal muscular atrophy [141]. Conversely, systemic intravenous delivery in patients with acute stroke [135] and multiple sclerosis [140] is safe but is not associated with significant clinical trends. This limited efficacy primarily arises from pulmonary cell entrapment, which prevents cells from crossing the complex biological barriers present in patients with advanced neurodegenerative conditions.

Crucially, a cell's specific tissue origin serves as a key determinant of neurological therapeutic efficacy. Umbilical cord‐derived stem cells, for instance, generate dopaminergic neuron‐like cells more efficiently than their adipose‐derived counterparts [166]. Microenvironmental preconditioning yields distinct secretomic signatures. Priming AD‐MSCs with hair follicle stem cell medium selectively increases nerve growth factor synthesis, distinguishing them from marrow‐derived populations, in which the expression of brain‐derived neurotrophic factor is increased [165, 167]. Elucidating these intrinsic transcriptomic differences enables the precise selection of specialized cellular subpopulations for targeted neurological repair [168, 169].

Similarly, within the ophthalmic microenvironment (Figure 2G), AD‐MSCs exert potent immunomodulatory and regenerative effects. AD‐MSC‐derived EVs carry abundant protective bioactive molecules to facilitate ocular tissue repair and preserve endogenous limbal stem cell populations through paracrine signaling. These cells are genetically modified to overexpress SPARC [114]. Reconstructing the depleted limbal niche requires rigorous sequential induction, where the initial inhibition of the TGF‐β and Wnt pathways drives surface ectoderm formation. Subsequent culture with specific growth factors on a vitronectin matrix results in the successful generation of limbal stem cell‐like cells [170]. Transplanting AD‐MSCs onto denuded amniotic membranes facilitates their targeted binding to inflamed regions, suppressing pathological angiogenesis to restore cytokeratin 3 (CK3) and p63 expression [113]. Ex vivo priming in specialized medium further optimizes this paracrine profile by decreasing the secretion of VEGF and MMP‐2. Following in vivo engraftment, this optimized secretome increases IL‐10 levels and preserves corneal transparency [111]. Intracameral electroporation of modified EVs alleviates oxidative stress and cellular senescence and blocks the endothelial–mesenchymal transition (EndoMT), thereby preventing irreversible corneal tissue injury [112]. This effective acellular immunomodulation extends the therapeutic benefits to resolving retinal ischemia and diabetic retinopathy [171].

### 4.5 AD‐MSCs for Cardiovascular Diseases: Mechanisms, Clinical Evidence, and Translational Challenges

The primary therapeutic mechanisms of AD‐MSCs in cardiovascular diseases involve promoting angiogenesis and myocardial repair to restore cardiac function (Figure 2C). Through pleiotropic paracrine signaling, AD‐MSCs secrete proangiogenic and immunomodulatory factors to remodel the injured myocardial microenvironment, accelerating cardiac tissue repair, and functional recovery [172]. Specifically, AD‐MSC‐EVs deliver diverse bioactive molecules that upregulate the expression of genes involved in the mitochondrial respiratory chain complex (such as *MT‐ND1* and *MT‐CO1*) in recipient endothelial cells, promoting proliferation and angiogenesis [172, 173]. Furthermore, AD‐MSCs mitigate myocardial inflammatory responses by suppressing the expression of proinflammatory mediators such as TNF‐α and IL‐6 [174, 175]. In the context of atherosclerosis, encapsulating AD‐MSCs within Type I collagen microspheres downregulates ICAM‐1 and VCAM‐1 expression, suppressing the EndMT and attenuating atherosclerotic progression [176].

Clinical evaluations reveal critical delivery barriers. An intracoronary infusion of uncultured AD‐MSCs ameliorates myocardial perfusion deficits and reduces the infarct volume in patients with acute myocardial infarction [142]. Intramyocardial implantation of the SVF directly targets akinetic myocardial scars. By avoiding cell expansion ex vivo, these localized injections improve the ejection fraction and preserve exercise capacity, suggesting a potential to reduce the risk of end‐stage heart failure [121]. Additionally, targeted intra‐arterial delivery into stenotic renal arteries dampens local inflammatory responses and revascularizes hypoxic tissues [137]. Despite these exploratory validation experiments, a translational discrepancy persists between the preliminary results from Phase I trials and the definitive primary endpoint failures characterizing recent Phase II/III RCTs. This failure highlights a major conceptual flaw in the historical literature: researchers mistakenly assumed that heavily cultured cells would act exactly like uncultured tissue fractions. Early clinical milestones, such as the APOLLO trial [142], demonstrated that the intracoronary administration of uncultured AD‐MSCs significantly reduced the infarct size in patients with acute myocardial infarction. Subsequent multicenter RCTs, however, specifically the SCIENCE [177] and DANISH [178] trials, utilized fully culture‐expanded allogeneic AD‐MSCs and did not observe significant improvements in the left ventricular ejection fraction (LVEF) in chronic ischemic heart failure cohorts [179].

This translational barrier necessitates a critical reappraisal of the underlying paracrine hypothesis and the predictive validity of preclinical models. The therapeutic efficacy of a cell depends on how long it survives and the specific environment it faces. These conflicting outcomes do not indicate intrinsic limitations in the cellular therapeutic capacity; rather, they reflect a mismatch between cellular pharmacokinetics and the pathological recipient microenvironment. Uncultured AD‐MSCs natively retain their pericytic epigenetic imprinting, while the acute ischemic microenvironment concurrently provides necessary reciprocal immune licensing. In contrast, extensive in vitro expansion systematically downregulates the expression of specific chemokine receptors, particularly CXCR4, thereby impairing the directional homing capacity [180]. Furthermore, the chronic heart failure microenvironment presents an avascular, highly crosslinked, and fibrotic hostile matrix. When engrafted into this severely scarred tissue, expanded cells face a profound lack of topographical ECM integrin‐binding sites. This impairment of mechanotransduction may rapidly induce anoikis (detachment‐induced apoptosis) in a large proportion of cells [181], leading to accelerated reticuloendothelial clearance before sufficient paracrine concentration gradients can be established [182]. Standard murine acute ligation paradigms utilize young, metabolically resilient subjects and fail to replicate the complex pathophysiological burden characterizing ischemic heart failure in humans. Human pathologies are compounded by advanced chronological age, systemic metabolic comorbidities, and extensive polypharmacy that actively antagonize MSC activation. This biological mismatch renders traditional acute preclinical outcomes poorly predictive of chronic efficacy in humans [183].

Finally, the reliance on the LVEF or end‐systolic volume as primary clinical endpoints requires a careful re‐evaluation. Once a heart has developed thick fibrotic scars, no therapy can fundamentally reverse the established macroscopic structural damage. Accumulating mechanistic evidence indicates that AD‐MSCs promote microvascular repair and lower local inflammation but do not generate large volumes of myocardial tissue de novo. Consequently, the LVEF lacks the sensitivity to detect subtle microvascular and antifibrotic therapeutic effects. Future trial designs should prioritize functional and patient‐centered outcomes, such as regional myocardial strain, microvascular perfusion mapping, decreases in major adverse cardiovascular events (MACEs), or improved quality‐of‐life assessments, to more accurately capture the full clinical utility of AD‐MSCs [184, 185].

### 4.6 Respiratory and Urogenital Fibrotic Pathologies

Current therapeutic strategies for pulmonary fibrosis prioritize functional preconditioning rather than direct cell replacement (Figure 2D). Preconditioning stromal cells with low‐molecular‐weight heparin activates the SDF‐1/CXCR‐7 axis, increasing the secretion of hepatocyte growth factor (HGF). This modification promotes targeted homing to injured lung tissue, allowing the cells to exert antifibrotic effects at reduced fractional doses via the inhibition of IL‐6 secretion [115, 116, 186]. The restorative mechanisms adapt to specific microenvironmental stressors. Cellular grafts undergo the MET to reconstruct the alveolar architecture in patients with emphysema [117] and reduce early neutrophil infiltration to ameliorate acute respiratory distress [118].

In clinical studies, an intravenous infusion of allogeneic AD‐MSCs is a well‐tolerated treatment for acute respiratory distress syndrome. This intervention limits alveolar epithelial injury, with a corresponding downregulation of surfactant protein D expression [136]. An analysis of the intracellular secretome revealed the upregulation of protein tyrosine phosphatase receptor type R (*PTPRR*) in pulmonary fibroblasts. Through the dephosphorylation of the extracellular signal‐regulated kinase cascade, this enzyme directly inhibits the transcription of ACTA2 (α‐SMA) and collagen. Targeted treatment reversed pathological matrix deposition in humanized xenografts, even in xenografts from individuals with advanced disease stages, providing preclinical evidence for the treatment of established fibrosis [116].

Fibrosis in conditions such as urethral stricture primarily manifests as excessive collagen deposition and chronic inflammatory responses (Figure 2E). Integrating EVs into an electrospun collagen and polycaprolactone nanoyarn scaffold yields mechanical support for tissue regeneration while maintaining sustained paracrine signaling. This composite biomaterial polarizes local macrophages toward an anti‐inflammatory CD206‐positive phenotype. This macrophage phenotypic shift suppresses IL‐6 secretion and halts aberrant Type I collagen accumulation, inducing the regeneration of functional vascular networks and the stratified urothelium [119].

The treatment of complex long‐segment urethral defects typically requires multimodal therapeutic approaches. Treatment with progenitors seeded onto functionalized graphene oxide (GO/PEG‐NH_2_/PCL) nanostructures along with oral sildenafil administration exerts antifibrotic effects. Sildenafil utilizes the cyclic guanosine monophosphate pathway to downregulate MMP2 expression, eliminate severe tissue scarring, and restore physiological uroplakin and desmocollin levels to achieve anatomical recovery [120]. Interestingly, when treating stress urinary incontinence, a systemic intravenous infusion of EVs often confers a greater therapeutic benefit than a local injection. This difference is likely attributable to the broader tissue distribution of therapeutic bioactive factors after intravenous delivery [187].

In addition to urological pathologies, paracrine modulation has renoprotective effects. Weight‐based dosing leverages systemic immunomodulatory pathways, limiting autoreactive T‐cell proliferation and restoring immune homeostasis in individuals with conditions such as recent‐onset type 1 diabetes [129, 130] and lupus nephritis [146], without increasing the risk of microvascular thrombotic events. The intravenous injection of allogeneic AD‐MSCs exhibits a favorable safety profile in the treatment of chronic kidney disease. Despite failing to reduce proteinuria significantly, systemic therapy stabilizes the short‐term decrease in the glomerular filtration rate and slows progressive renal deterioration compared with real‐world control treatments [131, 132]. Low‐dose administration results in the greatest stability, indicating that the reparative mechanism depends on endogenous repair rather than dose‐dependent engraftment [132]. Systemic administration also mitigates reproductive hormone deficiency, improving sexual quality of life, which highlights the rejuvenative potential of the adipose‐derived secretome [128].

## 5 Translational Bottlenecks: Addressing Heterogeneity, Safety Risks, and Poor Engraftment

While the clinical applications outlined above indicate the promising therapeutic potential of AD‐MSCs, moving these therapies from the laboratory into routine medical practice remains a substantial challenge. We systematically examine the core biological barriers impeding effective clinical translation to determine the reasons for the inconsistent outcomes reported in early clinical trials. In the following sections, we dissect three core roadblocks: the inherent heterogeneity of cell batches, underlying tumorigenic and safety concerns, and rapid donor cell death following in vivo administration. Finally, we explore how optimized cell preconditioning and precise CRISPR engineering may help mitigate these limitations.

### 5.1 Multidimensional Heterogeneity

Contemporary regenerative biology attributes AD‐MSC transcriptomic heterogeneity to four primary determinants: the anatomical tissue location, donor metabolic stress burden, ex vivo phenotypic drift, and inherent subpopulation diversity.

Governed by divergent embryonic origins, tissue‐specific niches shape distinct baseline phenotypes via site‐specific mechanotransduction cues. The osteochondral plasticity of the infrapatellar fat pad, for example, is superior to that of subcutaneous fat pads, which are enriched for distinct proangiogenic subclusters [188, 189, 190]. Systemic physiological and metabolic stressors remodel the epigenetic profile of donor AD‐MSCs. In the context of active malignancy, omentum‐derived progenitor cells undergo aberrant osteogenic differentiation, whereas their subcutaneous adipose‐derived counterparts are skewed toward a proinflammatory secretory phenotype [191]. This systemic allostatic burden introduces variability, skewing the derived vesicular secretomes toward origin‐dependent therapeutic trajectories: adipose‐derived EVs favor immunomodulation over structural repair, whereas bone marrow‐derived and umbilical cord‐derived EVs specialize in structural repair [192, 193, 194, 195].

Extrinsic phenotypic variability emerges during ex vivo propagation. Transferring these progenitors from a compliant 3D perivascular niche to rigid 2D culture surfaces imposes strong mechanical selective pressure. Such sustained in vitro stress accelerates replicative senescence across cell subpopulations. Strategies for mitigating this culture‐induced change in function include preserving immunomodulatory function using immortalized cell lines [196] or enriching adipogenic subpopulations using label‐free dielectrophoresis [197] and targeted magnetic sorting [42].

Addressing the inherent cellular heterogeneity within a single batch remains a central challenge in the field. High‐resolution single‐cell transcriptomics enables the construction of detailed maps of these functional subpopulation hierarchies, yet integrating such multiomic platforms into standard cGMP workflows remains logistically and economically prohibitive for real‐time batch release. In practice, this regulatory constraint forces researchers to focus on scalable physicochemical quality control assays. High‐throughput liquid chromatography–tandem mass spectrometry characterizes bulk‐cell batches based on dominant secretome profiles and identifies distinct EV‐associated ribosomal proteins that distinguish regenerative cells from proinflammatory cells [198, 199]. Because analytical qualification structurally fails to physically deplete antagonistic cells, scalable manufacturing increasingly integrates label‐free biophysical separation. Advanced secretion‐encoded single‐cell sequencing identifies rare super‐secretors but functions exclusively as an upstream discovery engine because of limitations in scalability [200]. Conversely, microfluidic platforms based on continuous‐flow deterministic lateral displacement separate functional cellular subsets by exploiting their inherent morphological differences [201]. This physical sorting avoids harsh chemicals, preserves the native genomic integrity, and adheres to minimal manipulation principles, resulting in the transformation of a random mixture of cells into a highly engineered precision drug.

### 5.2 Clinical Safety and Oncological Risks

Evaluations of the clinical safety of AD‐MSCs traditionally focus on acute adverse immune reactions and the potential for malignant transformation. Historically, MSCs have been widely regarded as having low immunogenic potential due to their lack or low expression of major histocompatibility complex (MHC) Class II molecules and key costimulatory ligands [179, 202]. Although matching for major HLA antigens prevents acute, organ‐level rejection, minor differences in amino acid levels still cause subclinical immune reactions. Ultimately, the patient's immune system recognizes these allogeneic cells and mediates their rapid clearance. This elimination process can also induce the formation of donor‐specific antibodies. This allosensitization presents formidable antibody‐mediated rejection risks for patients awaiting future solid organ transplants [203], necessitating stringent immunological profiling prior to administration.

The reliance on the absence of spontaneous malignant transformation as the sole metric of oncological safety represents a critical oversight in risk assessments [204, 205, 206]. Unmodified AD‐MSCs lack an intrinsic tumorigenic capacity, yet their immunomodulatory secretome exerts dual effects in postresection tumor microenvironments. Via tumor‐stromal crosstalk, residual malignant cells can epigenetically subvert naive AD‐MSCs into a cancer‐associated phenotype. This phenotypic transition supports the formation of a permissive premetastatic niche through extensive ECM remodeling and the induction of immune evasion mechanisms [207].

These interactions occur via distinct mechanistic layers. At the intact cellular level, a direct interaction facilitates horizontal mitochondrial transfer into breast cancer cells through tunneling nanotubes [208]. This bioenergetic input promotes metabolic reprogramming and the acquisition of multidrug‐resistant phenotypes in malignant cells [208]. At the cell‐free level, native EVs transfer specific noncoding RNAs, notably miR‐23b, to induce a dormant state in recipient tumor cells [209]. This transfer confers latent chemoresistance and generates dormant tumor cell populations that represent a source of subsequent disease relapse. However, this interaction remains unpredictable. Precisely fractionated EV subsets have the ability upregulate proapoptotic gene networks in identical tumor models [210]. This dual mechanistic profile necessitates clear contraindication criteria and oncological monitoring for patients with a prior malignancy who are receiving AD‐MSC‐based therapies. When these immunological and oncological risk considerations are appropriately addressed, long‐term follow‐up shows that AD‐MSCs have a favorable safety profile and support sustained tissue repair, with side effects that are predominantly mild and related to the injection procedure [211, 212, 213, 214, 215, 216].

### 5.3 The Transdifferentiation Controversy

For decades, researchers in the regenerative medicine field have widely believed that AD‐MSCs function by structurally replacing damaged tissue. Today, advanced biology has overturned this long‐standing theory. Orthogonal dual‐recombinase lineage‐tracing systems have provided definitive evidence against cross‐germ layer transdifferentiation in vivo [217]. Earlier studies utilizing generic lipophilic dyes revealed that recipient phagocytes that engulf apoptotic cell debris can be misidentified as engrafted donor cells in ischemic tissue microenvironments [218]. Additionally, rare observations of apparent cell fate conversion have been attributed to spontaneous cell fusion events rather than genuine transdifferentiation.

The rapid death of donor AD‐MSCs in vivo does not represent a failure of therapeutic efficacy. Rather, it constitutes the mechanistic basis underlying their therapeutic effects. Immunology currently recognizes rapid stromal clearance as an active, recipient‐mediated cytotoxic licensing event. Recipient lymphocytes release perforin and granzyme to induce AD‐MSC apoptosis upon engraftment [219]. Professional phagocytes rapidly internalize these apoptotic remnants via efferocytosis, resulting in transcriptional reprogramming that compels recipient macrophages to polarize toward a proresolving M2 phenotype [218, 219]. Transient colonization, consequently, serves as the key mechanistic trigger for restoring systemic tissue homeostasis.

Within this apoptosis‐driven framework, achieving local therapeutic concentrations before immune clearance requires the optimization of delivery pharmacokinetics (Table 5). Intravenous administration consistently results in pulmonary sequestration; nevertheless, this initial pulmonary retention drives the systemic release of immunomodulatory cytokines prior to cell death [220]. Localized injections bypass systemic attrition but risk an uneven spatial distribution and regional inflammatory reactions. Interventional cell delivery reduces the cell dose required to achieve therapeutic efficacy and lowers the risk of microvascular thromboembolism and local tissue injury [221].

**TABLE 5:** Comparative summary of the administration routes for AD‐MSCs in vivo.

Administration route	Advantages	Disadvantages	Refs.
Intravenous infusion	Pulmonary sequestration can strategically trigger widespread systemic immunomodulation and the broad suppression of proinflammatory cascades prior to cellular exhaustion. Exhibits a highly favorable short‐term safety profile with no evidence of systemic inflammatory spikes, anomalous neoplastic proliferations, or adverse vital physiological alterations. Offers a minimally invasive workflow with high reproducibility and convenience.	The pulmonary first‐pass effect causes systemic cellular attrition and highly restricted spatial engraftment at remote injury sites, diluting localized therapeutic potency. The inability to bypass systemic dilution prevents the establishment of the robust localized paracrine gradients necessary for addressing focal structural defects.	[220, 221]
Localized injection	Effectively circumvents systemic circulatory dilution and pulmonary entrapment barriers, ensuring maximal initial cell loading directly within the pathological niche. Facilitates the establishment of highly concentrated localized paracrine gradients.	Forcing cells directly into hostile or inflammatory microenvironments accelerates apoptotic clearance and metabolic exhaustion. Risks triggering a pronounced localized inflammatory shock, significantly increasing focal levels of inflammatory markers, and may induce secondary mechanical injury due to the concentrated cellular bolus. Severely limits widespread intraorgan distribution, rendering it unsuitable for diffuse parenchymal diseases.	[221]
Interventional delivery	Synergizes the high‐efficiency targeting of localized injections with the widespread organ distribution of systemic infusions, effectively bypassing pulmonary sequestration. Lowers the requisite cellular dose to achieve therapeutic efficacy, thereby diminishing the risks of microvascular thromboembolism and localized tissue trauma. Safely mitigates local inflammation and restores physiological perfusion in targeted organs without provoking the regional inflammatory shock observed with direct local injections.	Inherently restricted by a steep technical learning curve, necessitating specialized angiographic equipment, contrast agents, and invasive catheterization protocols. Although highly selective, minor fractions of the cellular payload may still evade local capillary capture and leak into the systemic circulation, albeit at negligible levels compared to the IV route.	[221]

The recipient microenvironment critically modulates transient retention, utilizing injury‐induced chemokines to direct CXCR4‐dependent migration alongside localized WNT and NF‐κB signaling cascades to drive functional activation [222, 223, 224]. The tissue origin of AD‐MSCs further influences their homing capacity. Compared with their subcutaneous counterparts, peripancreatic adipose‐derived subsets exhibit accelerated inflammatory resolution [73]. Because unencapsulated free cells readily undergo apoptosis in ischemic tissue microenvironments, overcoming this pharmacokinetic limitation requires precision delivery. Researchers are currently rewiring AD‐MSCs through targeted genetic modifications and synergizing these engineered cells with biomimetic hydrogels [114, 225]. This combined approach provides physical protection for cells, decelerates apoptotic kinetics, and extends the spatiotemporal window of paracrine factor release.

### 5.4 Strategies for the Targeted Preconditioning of Functional Cellular Subpopulations

Researchers typically use biochemical priming and biophysical cues to increase therapeutic efficacy before engraftment [73, 226]. Evaluating these conventional methods, however, reveals translational limitations. The exposure of unsorted bulk‐cell populations to acute metabolic stress or supraphysiological inflammatory licensing causes excessive cellular stress and frequently impairs endogenous antioxidant defenses.

Functional cellular subpopulations exhibit heterogeneous stress tolerance profiles [14, 15, 227]. Effective preconditioning induces metabolic reprogramming toward aerobic glycolysis to support secretory effector function [228]. For cells derived from donors with metabolic comorbidities, uncalibrated ex vivo stimuli exceed the cellular metabolic reserve capacity, driving mitochondrial network remodeling and excessive reactive oxygen species accumulation. Although additional in vivo studies are needed to fully characterize the effects of these stressors on isolated cellular populations, in vitro findings consistently indicate associated risks. Overstimulating compromised progenitor cells may drive them toward premature replicative exhaustion [229]. Driven by dysregulated stress‐responsive kinase signaling pathways, stress‐induced premature senescence (SIPS) results in the secretion of deleterious paracrine mediators that impair regenerative efficacy [230].

Mitigating these non–cell‐autonomous effects requires recognizing that senescence‐associated phenotypes depend on the tissue of origin. Comparative omics analyses have demonstrated tissue‐specific functional divergence, as senescent bone marrow‐derived secretomes promote local adipogenesis, whereas adipose‐derived senescent cell secretomes suppress the regenerative plasticity of adjacent progenitor cells [231]. Furthermore, EVs secreted by metabolically exhausted AD‐MSCs propagate this senescent phenotype to naive recipient cells by driving aberrant nuclear signaling [232]. These depot‐specific functional differences invalidate the use of conventional uniform bulk‐cell preconditioning strategies. Preserving therapeutic potency requires the resolution of transcriptomic heterogeneity at the single‐cell subpopulation level. Integrating metabolic checkpoints, notably TIGAR‐mediated glycolytic regulation [233], with targeted senotherapeutic interventions to transiently clear exhausted subclusters during culture expansion has emerged as a key strategy for next‐generation biologics [234].

### 5.5 Posttransplantation Epigenetic Drift and Phenotypic Convergence

The delivery of highly purified cellular subpopulations does not prevent postengraftment microenvironmental stress from altering their functional state. Acute ischemic and inflammatory tissue microenvironments impose critical physiological stress that disrupts ex vivo‐acquired therapeutic phenotypes, forcing rapid epigenetic drift and phenotypic convergence toward dysfunctional stromal lineages [235, 236].

Mechanistically, metabolic‐epigenetic crosstalk underlies this therapeutic attrition [237]. Unlike physiological hypoxia, severe pathological ischemia and oxidative stress precipitate the acute intracellular depletion of α‐ketoglutarate (α‐KG) and pathological accumulation of succinate and fumarate. This intermediate metabolite exhaustion competitively inactivates α‐KG‐dependent ten–eleven translocation (TET) dioxygenases and Jumonji C‐domain‐containing histone demethylases [238]. Concurrently, the failure of p62‐dependent autophagic clearance leads to the pathological accumulation of DNA methyltransferases (notably DNMT1/3A). Although this DNA methylation‐autophagy feedback loop was initially decoded in bone marrow stromal exhaustion [237], it exposes a highly conserved pan‐mesenchymal epigenetic vulnerability: this unsuppressed DNMT activity enforces aberrant de novo hypermethylation of the promoters of critical autophagic and cytoprotective genes. In AD‐MSCs, this chromatin reconfiguration induces severe epigenetic drift and SIPS, paralyzing the bioenergetic output required for mitochondrial transfer.

Pathological inflammation simultaneously rewires the histone code. Inflammatory stress leads to the marked downregulation of enhancer of zeste homolog 2 (EZH2), the catalytic subunit of polycomb repressive complex 2 (PRC2) [239]. Because healthy mesenchymal cells rely on this methyltransferase to deposit repressive H3K27me3 marks on senescence‐inducing loci, including CDKN2A, its stress‐induced decrease disrupts repressive epigenetic control. This process results in chromatin decondensation and the induction of the senescence‐associated secretory phenotype. Concurrently, histone deacetylase 9 (HDAC9) mediates the removal of active euchromatin marks such as H3K9ac. This chromatin remodeling blocks transcriptional access to regenerative gene promoters, resulting in loss of the ex vivo‐primed transcriptomic profile [240]. Unlike marrow‐derived progenitors, which develop an altered osteogenic‐adipogenic balance under chronic stress, adipose‐derived progenitors retain a distinct perivascular epigenetic signature [240]. These depot‐specific chromatin dynamics, therefore, represent a major biological barrier to maintaining the in vivo therapeutic efficacy of AD‐MSCs.

### 5.6 CRISPR‐Cas9‐Mediated Functional and Epigenetic Optimization of AD‐MSCs

The development of applicable off‐the‐shelf AD‐MSC therapeutics requires overcoming immunological and epigenetic barriers. cGMP protocols favor transient nonviral genome editing via the electroporation of Cas9 ribonucleoprotein (RNP) complexes. This modality circumvents the insertional mutagenesis inherent to canonical viral vectors, achieving homology‐directed repair while preserving genomic integrity [241].

Multiplexed genetic ablation subsequently targets key immunogenic loci to forge an immune‐stealth phenotype. Knocking out β‐2 microglobulin (β2M) alongside the Class II transactivator (CIITA) abolishes human leukocyte antigen expression on the cell surface, rendering engineered cells invisible to alloreactive T‐cell‐mediated clearance. Concomitant knock‐in of tolerogenic HLA‐E or HLA‐G molecules is required to evade natural killer (NK) cell‐mediated cytolysis. Additional knockout of the costimulatory ligands CD80, CD86, and CD40 further curtails dendritic cell maturation, synergistically allowing engineered cells to resist inflammatory tissue microenvironments [241].

In addition to structural immune evasion, catalytically inactive Cas9 (dCas9) systems enable targeted epigenetic modulation to counteract epigenetic drift in vivo. Fusing dCas9 to the TET1 catalytic domain forces targeted DNA demethylation at the promoters of anti‐inflammatory genes, including IL‐10 and TSG‐6 [242, 243]. This precise epigenome editing establishes a transcriptionally permissive state that resists hypoxia‐induced hypermethylation without altering underlying genomic sequences. Conversely, CRISPR interference deploying dCas9‐repressor fusion proteins enables the stable deposition of repressive heterochromatin marks at senescence‐associated and profibrotic genes.

The predictable clinical translation of this technology ultimately necessitates the integration of inducible safety switches to mitigate tumorigenic risks after permanent manipulation. The incorporation of suicide gene systems encoding inducible caspase‐9 (iCasp9) or herpes simplex virus thymidine kinase (HSV‐TK) established a tightly regulated feedback mechanism. A recent first‐in‐human Phase I trial using HSV‐TK‐engineered allogeneic AD‐MSCs for recurrent glioblastoma validated this safeguard, which ensured rapid apoptotic eradication of the engineered cells upon ganciclovir administration [244]. Integrating hypoimmunogenic editing with locus‐specific epigenetic stabilization increases long‐term cellular safety and functional stability, satisfying stringent regulatory mandates for advanced therapy and enabling the development of cell‐based therapeutic products.

### 5.7 A Mechanistic Cascade: Epigenetic Drift, Horizontal Mitochondrial Transfer, and Immunometabolic Reprogramming

The therapeutic efficacy of AD‐MSCs transcends the linear summation of isolated pathways, operating instead as a tightly integrated cascade of epigenetic, metabolic, and paracrine events. Microenvironmental stress initially triggers basal epigenetic priming. These chromatin alterations drive metabolic reprogramming, shifting progenitor cell metabolism toward aerobic glycolysis. Sustaining this metabolic phenotype enables AD‐MSCs to perform horizontal mitochondrial transfer by releasing EVs encapsulating noncoding RNAs and functional respiratory chain complexes. Ultimately, these vesicular signals enforce immunometabolic reprogramming within the recipient niche. Disrupting any node within this closed‐loop trajectory compromises therapeutic efficacy in translational settings.

At the molecular level, this coordinated cascade functions as an integrated epigenetic‐metabolic circuit. Recent evidence has established that oxygen‐ and α‐KG‐dependent dioxygenases, notably TET enzymes and Jumonji C domain‐containing histone lysine demethylases (KDMs), function as direct metabolic sensors. While physiological hypoxia preserves epigenetic integrity by restricting these oxygen‐dependent erasers to maintain chromatin integrity and delay senescence [238], pathological microenvironmental deterioration induces a distinct pathological stress response. Severe ischemia and oxidative bursts precipitate the acute depletion of the intracellular α‐KG pool alongside the pathological accumulation of succinate. This metabolic dysregulation competitively inhibits TET and KDM activities in a pathological context. Consequently, unsuppressed DNA methyltransferase (DNMT) activity is exacerbated by impaired p62‐dependent autophagic degradation, a highly conserved mechanism of epigenetic attrition that has recently been characterized across mesenchymal lineages [237], and drives aberrant de novo hypermethylation at the promoter regions of key autophagy‐related genes. In AD‐MSCs, this process structurally defines the epigenetic drift and premature senescence of the stromal compartment, which impairs the cellular bioenergetic capacity.

Functionally competent AD‐MSCs must overcome this epigenetic dysregulation to mediate intercellular mitochondrial transfer by shuttling intact respiratory chain complexes to compromised recipient cells to counteract this deterioration of the microenvironment and repair the tissue. This targeted metabolic support rapidly restores the recipient's mitochondrial membrane potential and mediates metabolic rescue. In damaged recipient macrophages, the restoration of mitochondrial OXPHOS reduces the intracellular accumulation of succinate, which acts as a critical danger signal that stabilizes HIF‐1α, drives glycolysis‐dominant M1 polarization, and reactivates the truncated tricarboxylic acid (TCA) cycle. By replenishing the α‐KG/succinate metabolic pool, mitochondrial transfer reverses the directionality of metabolic flux in recipient cells. This restored metabolic flux acts as an upstream regulatory signal, promoting nuclear epigenetic remodeling and shifting recipient macrophages to abandon the proinflammatory M1 state and commit strictly to an OXPHOS‐ and fatty acid oxidation‐driven M2 tissue‐reparative phenotype [245]. The integration of these interconnected mechanisms supports a unified mechanistic framework in which the preservation of AD‐MSC epigenetic integrity and autophagic–metabolic capacities is a fundamental prerequisite for mediating downstream immunometabolic tissue repair.

## 6 Conclusions and Perspectives

AD‐MSCs possess multilineage differentiation plasticity and pleiotropic immunomodulatory functions, conferring broad therapeutic potential across diverse pathological conditions. Traditional AD‐MSCs research has relied on unfractionated bulk populations, resulting in inconsistent outcomes and impeding clinical translation. We delineate the in vivo developmental origins and tissue niches of AD‐MSCs. Mounting evidence demonstrates that tissue spatial localization and embryonic lineage imprinting epigenetically shape multilineage plasticity and functional phenotypes, challenging the conventional reliance on unfractionated bulk populations. In ex vivo cell processing, we systematically evaluated a range of isolation and purification strategies, from enzymatic digestion to ultrasonic cavitation, to address major biomanufacturing bottlenecks. Through this comparative assessment of isolation methods, we consider magnetic‐activated cell sorting (MACS) as a key technology for the prospective stratification of functionally distinct cell subpopulations.

Turning to phenotype applications and clinical translation, this review synthesizes extensive preclinical and clinical evidence on the broad spectrum of pleiotropic therapeutic effects of AD‐MSCs and their EVs. We further systematically characterize their therapeutic activity across a wide range of pathological microenvironments, specifically including musculoskeletal tissue remodeling, cutaneous immunometabolic reprogramming, ophthalmic neuroprotection, cardiovascular angiogenesis, and antifibrotic effects in respiratory disorders. AD‐MSCs mediate tissue repair by remodeling injured microenvironments through a diverse array of immunometabolic reprogramming, potent antifibrotic signaling, targeted angiogenesis, intercellular mitochondrial transfer, and transient paracrine regulation. Localized delivery strategies have shown the clinical evidence.

Resolving the heterogeneous therapeutic outcomes across clinical translation efforts hinges on overcoming the core barriers limiting clinical progress: cellular heterogeneity, oncological and immunological safety concerns, the ongoing transdifferentiation debate, nonstandardized preconditioning regimens, culture‐induced phenotypic drift, and poor cell retention after transplantation. Conventional bulk‐cell study approaches often extrapolate in vitro findings to in vivo pathophysiological contexts, largely overlooking the metabolic and epigenetic vulnerabilities that drive cell functional exhaustion. Current evidence indicates that the in vivo fate of unmodified transplanted cells is driven primarily by rapid recipient‐mediated apoptotic clearance rather than sustained functional engraftment.

To mitigate the variability in therapeutic outcomes resulting from unstratified cell preparations and nonstandardized preconditioning protocols, drawing on the current evidence, we outline a refined framework for clinical translation centered on targeted solutions to key biological constraints. This framework follows a structured approach organized around three core focus areas: First, while our review highlights MACS for the prospective isolation of functional subpopulations, large‐scale manufacturing presents notable biomanufacturing challenges: hydrodynamic shear stress during continuous‐flow processing causes proteolytic cleavage of shear‐sensitive human surface receptors. Addressing this challenge requires precision gene editing to engineer shear‐resistant animal orthologs as stable surrogate sorting markers to enable scalable prospective isolation. Meanwhile, conventional rigid 2D monolayer expansion disrupts physiological mechanobiological properties via aberrant cytoskeletal tension, contributing to culture‐induced phenotypic drift. Reconstructing physiological tissue niches via mechanobiologically optimized 3D tissue‐mimetic biomaterials restores cellular metabolic homeostasis and improves posttransplant cell retention by providing a protective physical scaffold. Second, when combined with tailored preconditioning and precision CRISPR‐Cas9 epigenome editing to maintain an open euchromatin state at reparative gene loci, these structural and epigenetic modifications preserve transcriptomic identity. This effectively prevents epigenetic drift during automated closed‐system biomanufacturing, thereby preserving therapeutic potency and phenotypic stability. Third, expansion of therapeutic scope via cell‐free EV strategies. The inherent functional limitations, safety concerns, and hemocompatibility risks of living cells markedly limit their broad systemic application. This clinical application potential is realized through targeted EV engineering: distinct immunomodulatory EV subsets are purified via SEC, and functional modification of their lipid bilayers with tissue‐homing peptides confers improved tissue‐targeting specificity. Such targeted engineering approaches overcome biological barriers, allowing precise modulation of cell‐free therapeutic cargo to modulate and attenuate complex fibroinflammatory cascades across a wide range of organ‐specific pathologies.

Overall, this review provides more than a synthesis of evidence to support clinical translation of AD‐MSC therapies. The evidence compiled across this review demonstrates that successful clinical translation should be built on the prospective isolation of functional subpopulations, combined with tailored preconditioning, precision gene editing, 3D biomaterials, EV engineering, and automated closed‐system biomanufacturing. This integrated strategy is designed to preserve therapeutic potency, phenotypic stability, and sustained paracrine function, and support the transition of AD‐MSCs and their bioactive derivatives from variable, unfractionated preparations into well‐characterized, standardized, highly potent precision biologics. Furthermore, by systematically examining the AD‐MSCs research spanning from basic biology to clinical application, we integrate insights across multiple disciplines. Together, these concepts provide investigators across diverse disciplines and all stages of the research continuum with a unified theoretical framework, actionable guidance, and research inspiration, delineating a mechanism‐grounded, function‐driven, engineering‐enabled, and clinically accessible landscape for the advancement of next‐generation regenerative medicine.

## Author Contributions

**Ziyang Liu, Bailin Niu, and Li Chen**: conceptualization. **Ziyang Liu, Guangming Tu, and Ziyi Wu**: investigation. **Guangming Tu, Chao Wang, and Hongyan Zhao**: visualization. **Ziyang Liu**: writing – original draft. **Bailin Niu, Wei Li, and Li Chen**: writing – review and editing. **Bailin Niu**: funding acquisition. All the authors approved the final version.

## Funding

This work was supported by the Excellent Project of Chongqing Overseas Returnees' Innovation and Entrepreneurship Support Program [cx2025024], the Key Project of Science and Technology Planning of Chongqing Education Commission [KJZD‐K202600102], and the Chongqing Medical Scientific Research Key Project [Joint Key Project of Chongqing Health Commission and Science and Technology Bureau] (2023ZDXM012).

## Ethics Statement

The authors have nothing to report.

## Conflicts of Interest

The authors declare no conflicts of interest.

## Data Availability

The authors have nothing to report.
